# Longitudinal Cognitive Changes in Young Individuals at Ultrahigh Risk for Psychosis

**DOI:** 10.1001/jamapsychiatry.2018.1668

**Published:** 2018-07-25

**Authors:** Max Lam, Jimmy Lee, Attilio Rapisarda, Yuen Mei See, Zixu Yang, Sara-Ann Lee, Nur Amirah Abdul-Rashid, Michael Kraus, Mythily Subramaniam, Siow-Ann Chong, Richard S. E. Keefe

**Affiliations:** 1 Research Division, Institute of Mental Health, Singapore, Singapore; 2 Department of General Psychiatry 1, Institute of Mental Health, Singapore, Singapore; 3 Neuroscience and Behavioural Disorders, Duke-NUS Medical School, Singapore, Singapore; 4 Department of Psychiatry and Behavioral Sciences, Duke University Medical Center, Durham, North Carolina

## Abstract

**Importance:**

Cognitive deficits are a key feature of risk for psychosis. Longitudinal changes in cognitive architecture may be associated with the social and occupational functioning in young people.

**Objectives:**

To examine longitudinal profiles of cognition in individuals at ultrahigh risk (UHR) for psychosis, compared with healthy controls, and to investigate the association of cognition with functioning.

**Design, Setting, and Participants:**

This study has a multiple-group prospective design completed in 24 months and was conducted from January 1, 2009, to November 11, 2012, as part of the Longitudinal Youth at-Risk Study conducted in Singapore. Participants either were recruited from psychiatric outpatient clinics, educational institutions, and community mental health agencies or self-referred. Follow-up assessments were performed every 6 months for 2 years or until conversion to psychosis. Individuals with medical causes for psychosis, current illicit substance use, or color blindness were excluded. Data analysis was conducted from June 2014 to May 2018.

**Main Outcomes and Measures:**

Neuropsychological, perceptual, and social cognitive tasks; semi-structured interviews, and the Structured Clinical Interview for *DSM-IV* Axis I disorders were administered every 6 months. The UHR status of nonconverters, converters, remitters, and nonremitters was monitored. Cognitive domain scores and functioning were investigated longitudinally.

**Results:**

In total, 384 healthy controls and 173 UHR individuals between ages 14 and 29 years were evaluated prospectively. Of the 384 healthy controls, 153 (39.8%) were female and 231 (60.2%) were male with a mean (SD) age of 21.69 (3.26) years. Of the 173 individuals at UHR for psychosis, 56 (32.4%) were female and 117 (67.6%) were male with a mean (SD) age of 21.27 (3.52) years). After 24 months of follow-up, 383 healthy controls (99.7%) and 122 individuals at UHR for psychosis (70.5%) remained. Baseline cognitive deficits were associated with psychosis conversion later (mean odds ratio [OR], 1.66; combined 95% CI, 1.08-2.83; *P* = .04) and nonremission of UHR status (mean OR, 1.67; combined 95% CI, 1.09-2.95; *P* = .04). Five cognitive components—social cognition, attention, verbal fluency, general cognitive function, and perception—were obtained from principal components analysis. Longitudinal component structure change was observed in general cognitive function (maximum vertical deviation = 0.59; χ^2^ = 8.03; *P* = .01). Group-by-time interaction on general cognitive function (*F* = 12.23; η^2^ = 0.047; *P* < .001) and perception (*F* = 8.33; η^2^ = 0.032; *P* < .001) was present. Changes in attention (*F* = 5.65; η^2^ = 0.013; *P* = .02) and general cognitive function (*F* = 7.18; η^2^ = 0.014; *P* = .01) accounted for longitudinal changes in social and occupational functioning.

**Conclusions and Relevance:**

Individuals in this study who met the UHR criteria appeared to demonstrate cognitive deficits, and those whose UHR status remitted were seen to recover cognitively. Cognition appeared as poor in nonremitters and appeared to be associated with poor functional outcome. This study suggests that cognitive dimensions are sensitive to the identification of young individuals at risk for psychosis and to the longitudinal course of those at highest risk.

## Key Points

**Question:**

Do baseline and longitudinal cognitive architecture discriminate healthy controls from subgroups of young individuals at risk for psychosis?

**Findings:**

This multiple-group design study involving 384 healthy controls and 173 individuals at ultrahigh risk for psychosis found that baseline cognitive architecture differentiated healthy controls from converters and nonremitters. Remitters were found to recover their cognitive deficits over time, but nonremitters did not.

**Meaning:**

Cognitive deficits appear to identify the individuals most likely to develop psychosis and appear to reflect an underlying deterioration of a person’s clinical condition over time.

## Teaser

This longitudinal cohort study examines the trajectory of cognitive impairment in people younger than 30 years who are at the highest risk for developing psychosis.

## Introduction

Individuals who are prodromal to schizophrenia have a higher risk for and transition rate to psychosis compared with the general population.^1,2,3,4,5,6,7^ Cognitive deficits are also a predictor associated with psychosis.^3,7,8,9,10,11,12,13,14,15,16,17,18,19,20,21,22,23,24,25,26,27^ Cognitive impairments are the core disabling factors in psychosis and schizophrenia.^28,29,30,31^ Meta-analytic evidence indicates that cognitive deficits are present in individuals at ultrahigh risk (UHR) for psychosis.^24,25,26,32,33,34^ There is a 35% likelihood that the presence of symptoms—functional or cognitive manifestations—in high-risk, care-seeking individuals predates psychosis.^6^ However, systematic evidence is scarce for longitudinal cognitive trajectories in individuals at UHR for psychosis. Recent reports confirm that cognitive deficits at baseline are associated with conversion to psychosis, but the reports have not addressed the longitudinal cognitive profiles of these individuals.^27^ Equivocal evidence ranges from modest improvements in cognition in converters and first episode psychosis^26^ to suggestions that cognitive decline may be a strong factor in eventual psychosis.^33,35,36^ Previous reports indicate that approximately 50% of individuals at UHR for psychosis improve spontaneously within a short follow-up time frame.^37^

Longitudinal schizophrenia cognitive studies may offer insights to UHR cognitive trajectories. Premorbid cognitive deficits were found to be associated with schizophrenia.^33,38,39,40^ Cognitive impairment can be observed also in nonpsychotic family members of psychotic patients.^41,42^ Progressive changes in cognition over a 30-year period were reported in children who later developed schizophrenia.^33^ Two aspects of cognitive trajectories may be investigated: (1) means-based change, where differential time-based cognitive changes may exist between healthy individuals and those at UHR for psychosis, and (2) covariance-based change. The latter involves changes in the cognitive component structure, as defined by cognitive tests, over time^43,44^ and is known as the cognitive dedifferentiation hypothesis. This dedifferentiation is associated with poorer cognitive function with increased covariation across cognitive tests, a phenomenon previously observed in aging research.^43,44^ Intriguingly, forms of cognitive dedifferentiation were also noted in schizophrenia,^45,46^ where a subtle increase in test covariation was previously reported.^47,48^

We studied the prospective cognitive trajectories of individuals at UHR for psychosis. We expected to observe the greatest decline in cognitive performance over time among individuals at UHR who converted to psychosis compared with nonconverters and healthy controls. In individuals whose UHR status did not remit during the follow-up period, we expected to observe declining cognitive performance compared with remitters and healthy controls. We hypothesized that increased test covariance would be present as a function of time for individuals whose UHR status did not remit over time. Finally, we examined how changes in cognition as a function of time affected the social and occupational functioning of individuals at UHR for psychosis.

## Methods

Ethics approval for this study was provided by the Singapore National Healthcare Group's Domain Specific Review Board. Written informed consent was obtained from all participants, and consent from a legally acceptable representative was obtained for minors (younger than 21 years) as required by local regulations. This study was conducted from January 1, 2009, to November 11, 2012. Data analysis was conducted from June 2014 to May 2018.

### Participants

This study, as part of the Longitudinal Youth at-Risk Study conducted in Singapore,^49^ included 384 healthy controls and 173 individuals who met the criteria for UHR for psychosis.^12^ After 24 months, 383 healthy controls (99.7%) and 122 individuals at UHR for psychosis (70.5%) had remained in the study. Participants either were recruited from psychiatric outpatient clinics, educational institutes, and community mental health agencies or were self-referred. Individuals with neurological causes for psychosis, current illicit substance use, or color blindness were excluded. All participants were between 14 and 29 years of age. Their UHR status was ascertained by the Comprehensive Assessment of At-Risk Mental States,^12^ and their psychiatric history was evaluated with the Structured Clinical Interview for *DSM-IV Axis I Disorders*.^50^ Healthy controls did not fulfill UHR criteria, had no psychiatric disorder, and had no family history of psychosis.

Follow-up assessments at 6-month intervals for 2 years or until conversion to psychosis included the Positive and Negative Syndrome Scale,^51^ Beck Anxiety Inventory,^52^ Calgary Depression Scale for Schizophrenia,^53^ and the Social and Occupational Functioning Assessment Scale.^54^ Remission status was assessed at the 12- and 24-month time points. Individuals at UHR for psychosis were categorized into converters or nonconverters and remitters or nonremitters. Individuals at UHR at baseline but who no longer fulfilled UHR criteria at the 24-month time point were categorized as remitters. Those who met UHR criteria at final assessment or had converted to psychosis were categorized as nonremitters. In subsequent analyses, 2 sets of analysis were carried out involving (1) healthy controls, converters, and nonconverters and (2) healthy controls, remitters, and nonremitters. Details of the sampling methodology and the demographic characteristics of the sample were reported elsewhere.^49,55^

### Cognitive Measures

The Wechsler Memory Scale-III Spatial Span^56^; the Brief Assessment of Cognition in Schizophrenia,^57^ which consists of verbal memory, digit sequencing, token motor task, verbal fluency, symbol coding, and Tower of London tests; the Binocular Depth Inversion task^58,59^; the Continuous Performance Test—Identical Pairs^60^; the High-Risk Social Challenge skills interview^61^; the Babble task^62^; and the Snakes in the Grass test^63^ were administered. Cognitive tests were adjusted for age, sex, age × sex, age,^2^ and age^2^ × sex via linear regression modeling,^64^ and standardized residual scores were used for subsequent analysis. Cognitive scores were standardized against healthy control baseline measures. (See the Supplement for eAppendixes 1 and 2 [with eFigure 1], which deal with the concept of testing factor structure changes, and eAppendix 3 for data preprocessing details.)

### Statistical Analysis

Ordinal logistic regression was conducted to examine between-groups baseline cognitive differences. Univariate models that were *P* < .05 were selected for subsequent analysis. Linear mixed models were carried out to examine cognitive changes, allowing the inclusion of all longitudinal data available for each participant and the examination of the association of maturational stage with age-related trajectory changes over time. Stuart-Maxwell Marginal Homogeneity test was used to examine the divergence of the estimated test score distributions between the baseline and the 24-month follow-up for each group; these distributions were Bonferroni corrected. A principal components analysis (PCA) was conducted on baseline and 24-month cognitive batteries to investigate the cognitive component structure changes. Component loading vectors were compared via the Kolmogorov-Smirnov test (see eAppendixes 1 and 2 in the Supplement), and the comparisons were Bonferroni corrected. Cognitive components scores were compared using 1-way and repeated measures analysis of variance to examine group-by-time interactions. Repeated-measures general linear models were used to investigate the association of cognitive component changes with the rate of functioning changes. Bonferroni corrections for all intergroup comparisons were completed; further details of the data analysis are reported in eAppendix 3 in the Supplement. Analyses were conducted using SPSS, version 22.0 (IBM), unless otherwise noted.

## Results

### Demographics

Prospectively we evaluated 384 healthy controls (of whom 153 [39.8%] were female and 231 [60.2%] were male with a mean [SD] age of 21.69 [3.26] years) and 173 individuals at UHR for psychosis (of whom 56 [32.4%] were female and 117 [67.6%] were male with a mean [SD] age of 21.27 [3.52] years) who were between 14 and 29 years of age. Individuals at UHR for psychosis were further studied according to their conversion status (17 converters, of whom 3 [17.6%] were female with a mean [SD] age of 20.41 [3.18] years; 156 nonconverters, of whom 53 [34.4%] were female with a mean [SD] age of 21.37 [3.55] years) and remission status (84 remitters, of whom 28 [33.3%] were female with a mean [SD] age of 21.15 [3.41] years; 89 nonremitters, of whom 28 [31.5%] were female with a mean [SD] age of 21.38 [3.64] years). Further demographic characteristics are reported in the Table.

**Table.** Baseline Demographics Across Groups

Variable	Healthy Controls		Nonconverters		Converters^a^		Remitters		Nonremitters^a^	
	No.	Mean (SD)	No.	Mean (SD)	No.	Mean (SD)	No.	Mean (SD)	No.	Mean (SD)
Age, y	384	21.69 (3.26)	156	21.37 (3.55)	17	20.41 (3.18)	84	21.15 (3.41)	89	21.38 (3.64)
Female, %	153	39.8	53	34.4	3	17.6	28	33.3	28	31.5
Male, %	231	60.2	101	65.6	14	82.4	56	66.7	61	68.5
CAARMS total score	383	1.77 (3.67)	154	24.55 (15.57)	17	24.71 (11.09)	84	23.76 (14.79)	89	25.12 (15.43)
CDSS composite score	NA	NA	148	5.68 (4.86)	17	6.76 (5.97)	84	5.15 (4.62)	83	6.42 (5.21)
BAI composite score	NA	NA	146	19.97 (13.29)	17	23.65 (14.32)	82	18.57 (12.89)	83	21.78 (13.81)
PANSS total score	NA	NA	149	48.24 (11.62)	17	50.94 (12.90)	84	46.87 (11.44)	84	50.05 (11.78)
PANSS positive score	NA	NA	149	10.68 (2.79)	17	11.29 (3.06)	84	10.49 (2.75)	84	11.01 (2.84)
PANSS negative score	NA	NA	149	12.15 (4.24)	17	13.00 (3.61)	84	11.89 (4.37)	84	12.49 (3.97)
PANSS general psychopathology	NA	NA	149	25.41 (6.97)	17	26.65 (7.75)	84	24.49 (6.40)	84	26.55 (7.44)

Abbreviations: BAI, Beck Anxiety Inventory; CAARMS, Comprehensive Assessment of At-Risk Mental States; CDSS, Calgary Depression Scale for Schizophrenia; NA, not applicable; PANSS, Positive and Negative Syndrome Scale.

^a^ Converters are also part of the nonremitters.

No statistically significant differences in sex proportions were found across healthy controls, nonconverters, and converters (χ^2^_1_ = 3.74; *P* = .05) as well as healthy controls, remitters, and nonremitters (χ^2^_1_ = 3.74; *P* = .05). No statistically significant differences in age were observed among healthy controls, nonconverters, and converters (*F* = 1.53; *P* = .22) and healthy controls, remitters, and nonremitters (*F* = 1.02; *P* = .36). Statistically significant higher Comprehensive Assessment of At-Risk Mental State scores were observed in individuals at UHR for psychosis compared with healthy controls (*F* = 766.74; *P* < .001; η^2^ = 0.581). No notable differences were observed in the Positive and Negative Syndrome Scale, Calgary Depression Scale for Schizophrenia, and Beck Anxiety Inventory measures across groups (Table).

### Baseline Group Differences: Ordinal Logistic Regression

Baseline cognitive profiles of all tests are reported in eFigure 2 in the Supplement. Statistically significant between-group differences were found in verbal memory, digit sequencing, token motor task, verbal fluency, symbol coding, and Tower of London tests; the Wechsler Memory Scale-III Spatial Span; the High-Risk Social Challenge skills interview; the Snakes in the Grass test, and the Continuous Performance Test—Identical Pairs across groups (eTable 1 in the Supplement). Post hoc independent, unpaired, 2-tailed *t* tests revealed differences among healthy controls, nonconverters; healthy controls, converters; and healthy controls, nonremitters (eTable 1 in the Supplement). Baseline cognitive deficits were associated with psychosis conversion (mean odds ratio [OR], 1.66; combined 95% CI, 1.08-2.83; *P* = .04) and nonremission of UHR status (mean OR, 1.67; combined 95% CI, 1.09-2.95; *P* = .04).

### Cognitive Changes: Linear Mixed Models

**Figure 1.**
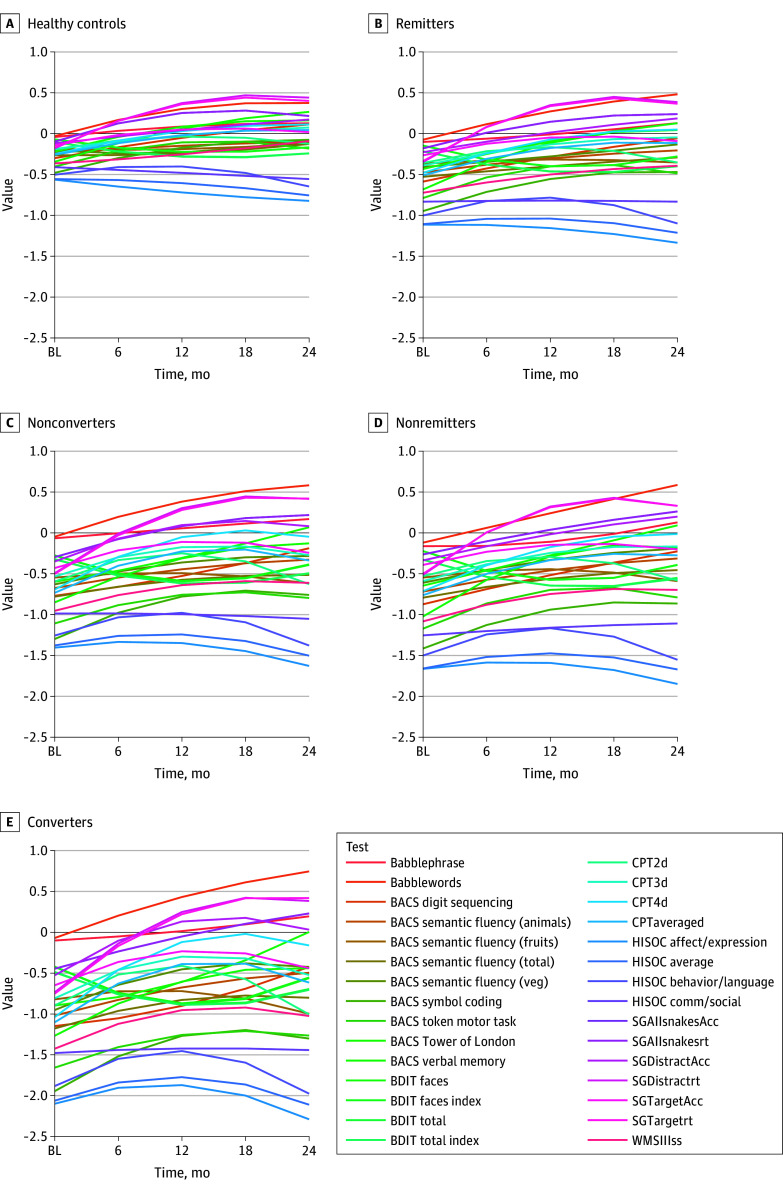
Cognitive Trajectories of Individual Tests Over 24-Month Follow-up A, Healthy controls. B, Remitters. C, Nonconverters. D, Nonremitters. E, Converters. Each test is color-coded. Individual lines reflect estimated cognitive scores for each test computed on the basis of linear mixed model outputs for each test. Babble indicates Babble Task; BACS, Brief Assessment of Cognition for Schizophrenia; BDIT, Binocular Depth Inversion Task; CPT, Continuous Performance Task: 2d, 2Digit, 3d, 3Digit, 4d, 4 Digit subtasks; HISOC, High Risk Social Challenge; SG, Snakes in the Grass test; Acc, Accuracy; rt, reaction time; and WMSIIIss, Wechsler Memory Scale – III Spatial Span.

Verbal memory, digit sequencing, token motor task, and symbol coding tests; the High-Risk Social Challenge skills interview; the Snakes in the Grass test; and the Continuous Performance Test—Identical Pairs showed longitudinal changes across all groups. Nonlinear changes in cognitive trajectories were also found (Figure 1; eTable 2 in the Supplement). Group-level differences were expected, but few group-by-time interactions were observed across cognitive tests (eTable 2 in the Supplement). The distributions of cognitive linear mixed models estimated scores were different at baseline and 24-month follow-up for all groups (eTable 6 in the Supplement). Increasing effect sizes across groups suggested that cognitive trajectories in nonremitters and converters were most divergent, pointing to subtle underlying perturbations of test covariation. Maturation stage (median split of age at baseline) and age-related trajectory changes were unremarkable. Statistically significant model changes were mostly due to the variability within the clinical groups rather than by observed group differentials (eFigures 3-9 and eTables 3-5 in the Supplement).

### Psychometric Architecture of Cognitive Constructs: Principal Components Analysis

**Figure 2.**
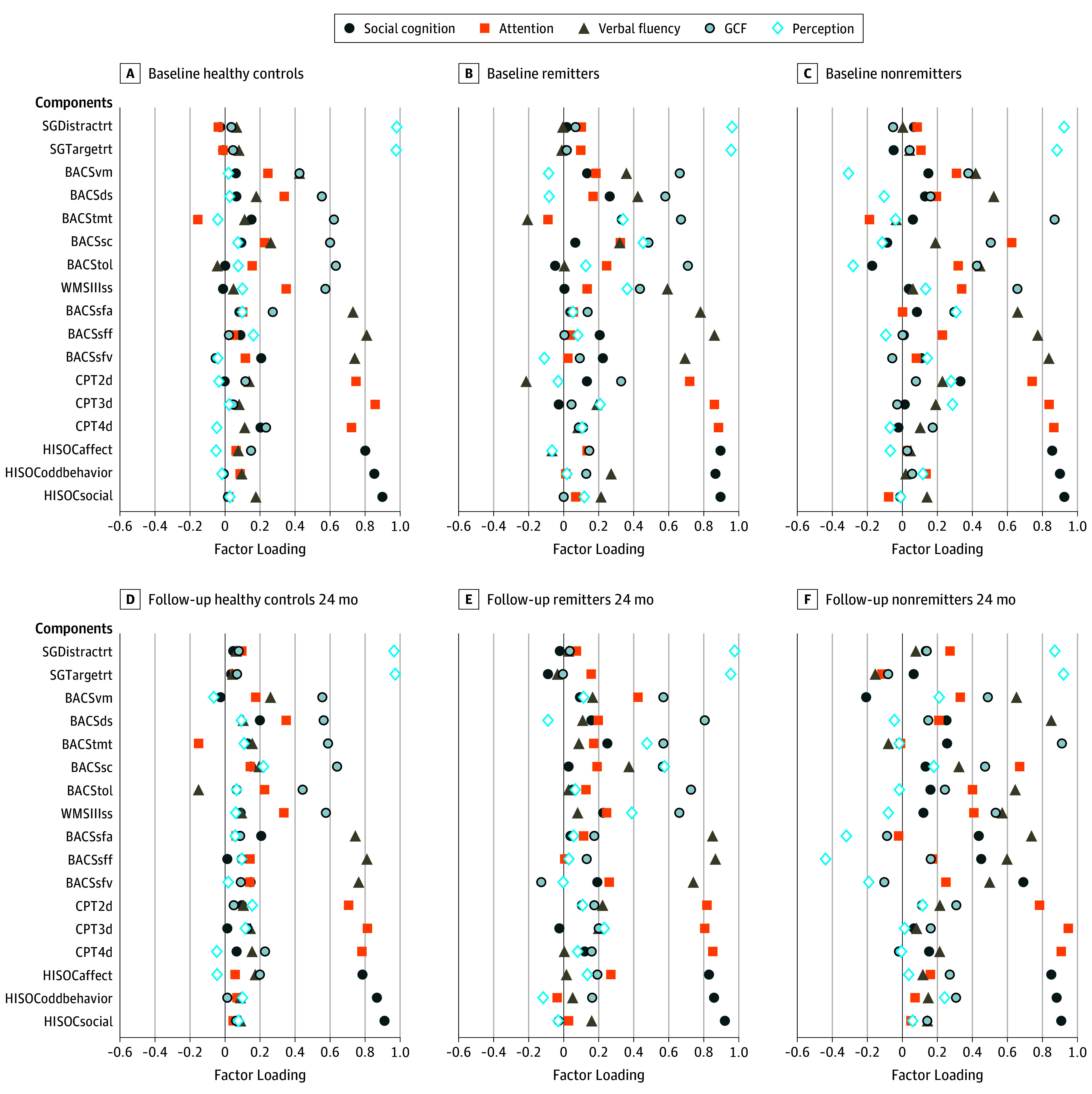
Component Loading Plots for Baseline and 24-Month Follow-up by Healthy Controls, Remitters, and Nonremitters A-C, Component loading plots for baseline. D-F, Component loading plots for 24-month follow-up. BACS indicates Brief Assessment of Cognition in Schizophrenia (ds, digit sequencing; sc, symbol coding; sfa, verbal fluency—animals; sff, verbal fluency—fruits; sfv, verbal fluency—vegetables; tmt, token motor task; tol, Tower of London; vf, verbal fluency; vm, verbal memory); Continuous Performance Task: 2d, 2Digit, 3d, 3Digit, 4d, 4 Digit subtasks; GCF, general cognitive function; HISOC, High-Risk Social Challenge; SG, Snakes in the Grass (Distractrt, distractor reaction time; Targetrt, target reaction time); WMSIIIss, Wechsler Memory Scale-III Spatial Span.

Twenty cognitive subtests with nominally significant (*P* < .05) baseline group differences were selected for PCA. Five orthogonal principal components were extracted using the Kaiser criterion^65^ (λ >1). Social cognition, attention, verbal fluency, general cognitive function (GCF), and perception were the 5 principal components that explained variances of 63.3% (healthy control), 74.1% (remitter), and 71.2% (nonremitter) at baseline and variances of 62.8% (healthy control), 75.7% (remitter), and 84.4% (nonremitter) at 24-month follow-up. The reliability of cognitive measures was comparable at baseline (overall α = .831; healthy control α = .792; remitter α = .845; nonremitter α = .809) and at 24-month follow-up (overall α = .848; healthy control α = .818; remitter α = .863; nonremitter α = .900). Component loadings by group and follow-up are represented in Figure 2.

Component load differences in GCF were noted among healthy controls, remitters, and nonremitters. Stark differences in component load between GCF baseline and 24-month follow-up in nonremitters were observed. Longitudinal changes for the component loadings for GCF in nonremitters were also observed (maximum vertical deviation = 0.59; χ^2^ = 8.03; *P* = .01). The observation is supported by results of the Kolmogorov-Smirnov tests that examined component load vectors across the PCA output (eTable 7 in the Supplement). Different load profiles were present among healthy controls, remitters, and nonremitters at baseline and at 24-month follow-up for the perception component, where subtler trends in social cognition load changes appeared in nonremitters but failed to survive the Bonferroni correction (eTable 7 in the Supplement).

### Longitudinal Change in Cognitive Constructs

**Figure 3.**
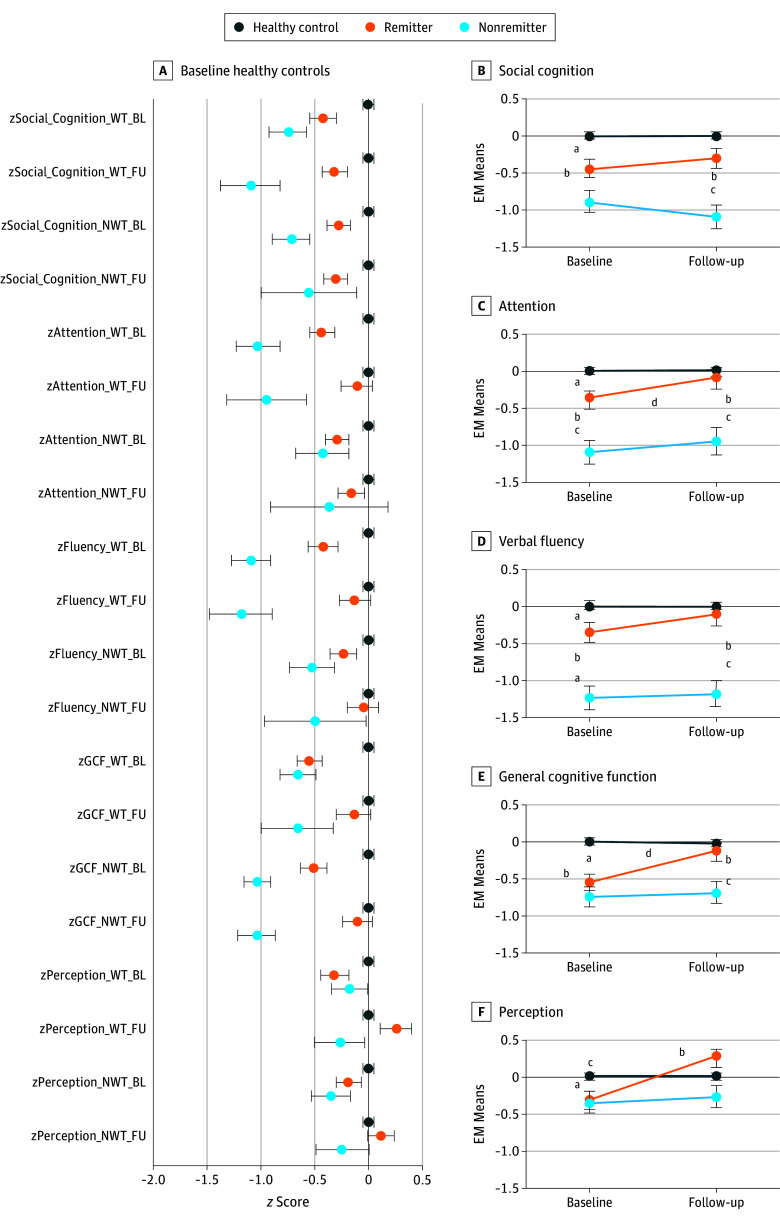
Cognitive Component Profiles by Group and Longitudinal Time by Group Models A, Cognitive component profiles by group. B-F, Longitudinal time by group. BL indicates baseline; EM, expectation maximization; FU, follow-up; GCF, general cognitive function; NWT, nonweighted; WT, weighted; z, standardized score. Bonferroni-corrected model significance is indicated by superscript notation. ^a^Healthy controls vs remitters. ^b^Healthy controls vs nonremitters. ^c^Remitters vs nonremitters. ^d^Remitters baseline vs remitters follow-up.

Weighted and nonweighted cognitive component scores were computed on the basis of the PCA results (eAppendix 3 in the Supplement). Repeated-measures analysis of variance was conducted on the cognitive component scores. Bonferroni-corrected α level of .025 was used to handle 2 test sets that evaluated the same hypothesis. Longitudinal changes were found for attention, GCF, and perception (Figure 3; eTable 8 in the Supplement). Post hoc Bonferroni-adjusted paired sample 2-tailed *t* tests showed improved performance in remitters, which accounted for the overall model effects. In nonweighted component scores, only GCF was found to be significant. Between-participant analysis of variance tests at both baseline and follow-up further confirmed that, although remitters appeared more similar to nonremitters at baseline for social cognition, attention, and GCF, their performance improved spontaneously with time, and remitters were more similar to healthy controls at 24-month follow-up (Figure 3; eTable 8 in the Supplement).

### Relation to Functioning

**Figure 4.**
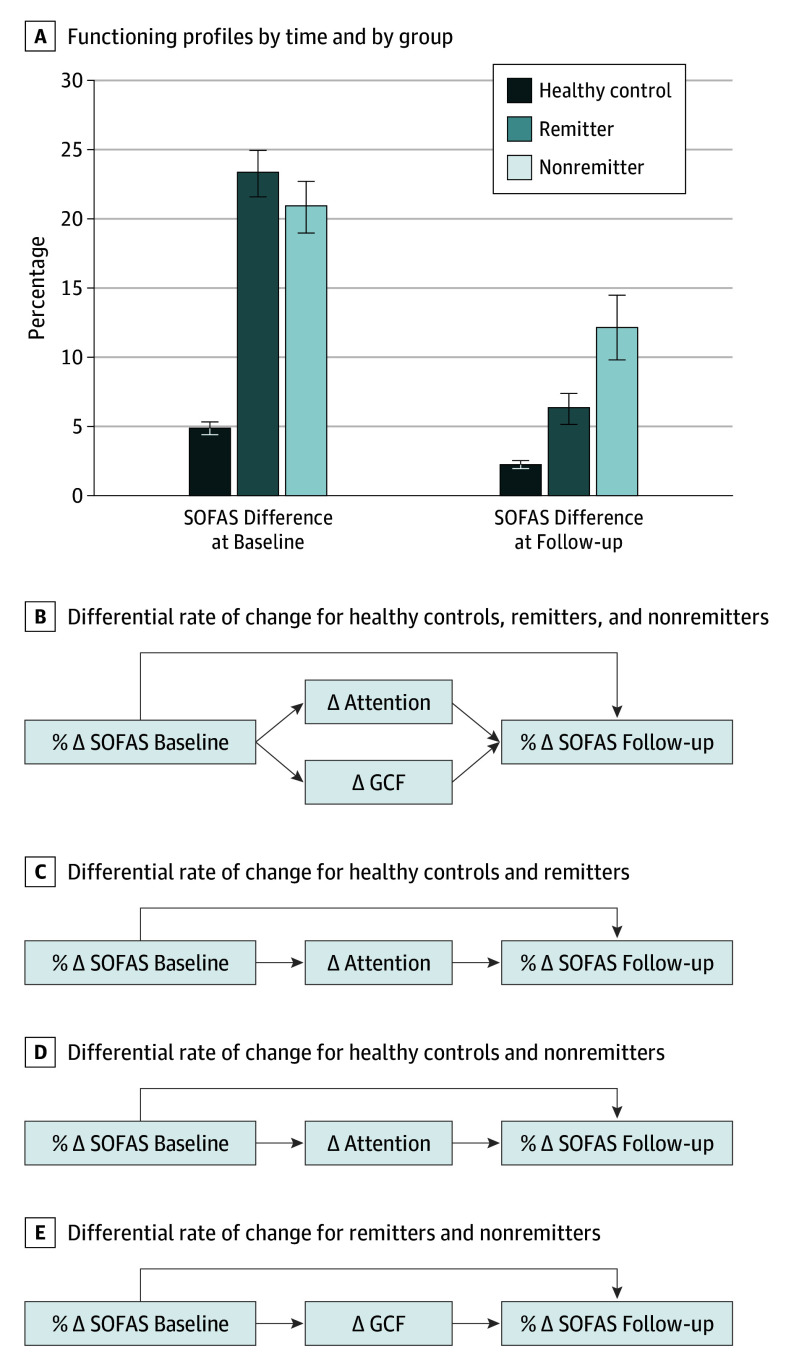
Social and Occupational Functioning and Cognitive Component Changes A, Functioning profiles by time point and group (healthy controls, remitters, and nonremitters). B, Repeated-measures schematics for time-by-group and time-by-cognitive component. Differential rate of change between baseline and follow-up: SOFAS: *F* = 36.85; *P* < .001; η^2^ = 0.130. Attention component: *F* = 5.65; *P* = .02; η^2^ = 0.013. GCF component: *F* = 7.18; *P* = .01; η^2^ = 0.014. C, Post hoc repeated measures. Differential rate of change between baseline and follow-up: SOFAS: *F* = 90.86; *P* < .001; η^2^ = 0.170. Attention component: *F* = 5.65; *P* = .02; η^2^ = 0.013. D, Post hoc repeated measures. Differential rate of change between baseline and follow-up: SOFAS: *F* = 8.67; *P* = .003; η^2^ = 0.020. Attention component: *F* = 15.83; *P* < .001; η^2^ = 0.036. E, Post hoc repeated measures. Differential rate of change between baseline and follow-up: SOFAS: *F* = 3.24; *P* = .07; η^2^ = 0.002. GCF component: *F* = 7.11; *P* < .009; η^2^ = 0.058. % indicates percentage difference between best and worst functioning during assessment time point; ∆, difference between baseline and follow-up; GCF, general cognitive function; and SOFAS, Social and Occupational Functioning Assessment Scale.

There was a main association of time with the range of social and occupational functioning at baseline and 24-month follow-up (Figure 4; eTable 9 in the Supplement). A statistically significant group-by-time interaction was observed, suggesting differential rates of change of functioning among healthy controls, remitters, and nonremitters. Group-by-time interaction on GCF (*F* = 12.23; η^2^ = 0.047; *P* < .001) and perception (*F* = 8.33; η^2^ = 0.032; *P* < .001) was present. Change in attention and GCF components appeared to partially mediate change in functioning (eTable 9 in the Supplement). Post hoc models revealed that change in the attention component (*F* = 5.65; η^2^ = 0.013; *P* = .02) partially mediated the spontaneous improvements in functioning in remitters and nonremitters compared with healthy controls (Figure 3E and Figure 4C and D). Change in GCF (*F* = 7.18; η^2^ = 0.014; *P* = .01) fully accounted for a differential rate of change in functioning between remitters and nonremitters. All post hoc comparisons survived Bonferroni correction.

## Discussion

To our knowledge, this study is the largest prospective single-site report of a case-control sample of individuals at UHR for psychosis. Comparisons between remitters and nonremitters suggested that above baseline cognition, trajectory and component-based analyses can identify psychosis and nonremission from illness. Worsening cognitive function over time may be a prime factor in eventual, if not incipient, psychosis.^33,36,66,67^

### Baseline Differences and Prediction Models

The study results are consistent with literature that shows significant cognitive deficits in UHR samples. Participants at UHR for psychosis were differentiated from healthy controls, and converters were differentiated from nonconverters according to baseline cognitive performance. Cognitive modeling results demonstrated statistically significant differences not only among healthy controls, converters, and nonconverters but also between individuals who met UHR criteria at baseline and those whose UHR status remitted, compared with those who had no remission.

### Prospective Trajectory Changes

Longitudinal modeling of cognitive performance revealed that most individuals improved with repeated testing every 6 months in the 24-month follow-up. Statistically significant group differences in trajectories were observed, suggesting that baseline variations in cognitive performance interact differently with time in the different groups. These results were consistent with earlier reports indicating that some individuals at UHR for psychosis display cognitive improvements with time.^67^ Practice effects, pharmacological effects, and diagnostic heterogeneity^67^ were alternative explanations for the phenomenon, but the more fine-grained follow-up neuropsychological test data reported here may offer further clarification of the cognitive trajectories of individuals at UHR for psychosis. Gradual increases in variability of test performance over time suggest the possibility that the underlying cognitive architecture may have devolved in converters and nonremitters during follow-up. Thus, measures of dedifferentiation of cognitive components may be 1 of the most powerful factors in later conversion and nonremission in individuals at risk for psychosis. Additional analysis of the maturational stage indicated that, between age 14 and 29 years, the most cognitive trajectory changes could be associated with clinical outcomes. Improvement of cognitive performance over time seems to be associated with age, but differential age-related cognitive trajectories do not appear to be present in groups at UHR for psychosis. Nevertheless, larger samples and wider age ranges might be required to further examine differential maturational profiles.

### Cognitive Architecture and Shifts in Component Loadings on Test Performance

Instead of maximizing the separation of cognitive components, we extracted them orthogonally to make apparent the cross-loading of cognitive subtests. Comparing PCA loading vectors revealed a significant shift in loading patterns between baseline and follow-up in nonremitters, implying the subtle changes in cognitive architecture over time. To our knowledge, such architectural changes have not been reported in previous studies of individuals at UHR for psychosis. We postulate that examining the prospective differential contribution of cognitive components to test performance could reveal subtle cognitive changes in at-risk states that will help differentiate between remitters and nonremitters. Covariance strength across cognitive test performance has been shown to yield vital insights into brain function in aging research^43,44,68^ and to be a property of deficit cognition in schizophrenia.^45,47,48^ Decreasing differentiation of GCF, perception, and social cognition components over time among nonremitters and converters is apparent.

### Investigating Cognitive Constructs

Cognitive components weighted by differential component loadings revealed more sensitivity with social and occupational functioning, particularly with the attention and GCF components. These findings indicate that incorporating cognitive architecture changes appears to be essential in uncovering subtle but important cognitive fluctuations that are relevant to functioning. Neither perception nor social cognition contributes to the variance in functional change beyond the traditional neuropsychological constructs. The trend related to the lack of clear group separation within the perception component could be attributed to the psychometrics of contributing tests. The Snakes in the Grass test, a visual search paradigm, may reflect the subtler changes in lower-level cognitive processes rather than the more robust separation in more traditional neuropsychological tasks. Nevertheless, the contribution of the perception component to test covariance supports the evidence that more refined cognitive mechanisms continue to be sensitive measures in identifying UHR for psychosis in general.^69,70,71,72^ Social cognition was the only construct that showed decrement over time in nonremitters beyond the baseline differences between healthy controls and remitters, although the component loading analysis suggested only trend-level dedifferentiation. It confirms that these findings replicate the notion that social cognition is separable from cognition even among individuals at UHR for psychosis.^73,74,75^ Longer follow-up periods might be necessary to determine whether an association between functioning and social cognition might emerge, similar to those in schizophrenia, as the downward trajectory in nonremitters ensues.^76,77^ Although speculative, mathematical models of cognitive architecture might be more sensitive than the standard neuropsychological tests to the changing neurobiology associated with emerging psychosis in young people at risk.

Cognition improved as a function of time, but the changes in remitters were dramatic. Remitters started at baseline with cognitive profiles that were similar to those of nonremitters, but their performance at follow-up was not different from that of healthy controls. The model that best exemplified this phenomenon included the measures comprising GCF. The correspondent longitudinal transition from dedifferentiation to differentiation of GCF that accounted for the functional recovery in remitters illuminates opportunities for follow-up work.

Overall, the results point to the possibility that UHR may not be a stable clinical or cognitive construct and that the deficits observed are transient. Results indicate that cognitive deficits in nonremitters tend to be stable and impaired in nearly all components. Longitudinal changes in cognitive architecture, particularly in remitters, have an association with the social and occupational functioning in young people. The converse could be true as the cognitive architecture continues to be increasingly dedifferentiated in nonremitters. These findings suggest that the prognosis for nonremitters is poor and will require the most clinical attention and remediation in the long term.

### Limitations

This study has several limitations. First, the conversion rate is low. Of the 173 participants at UHR for psychosis followed-up during a 2-year period, 17 (9.8%) converted to psychosis, which is a lower rate than most other reported conversion rates. We speculate that the reason may be the strict drug laws in Singapore and the structured nature of its society. Low conversion rate precluded more sophisticated analysis on convertors. Second, medication use was not systematically adjusted for in the current analysis. The individuals at UHR for psychosis were not medicated with antipsychotics, although some were taking antidepressants. The association of antidepressants with cognition was found to be weak,^78,79^ but no differences in anxiety or depressive symptoms among UHR groups were observed. Subsequent studies of psychotropic medications and their various cognitive outcomes in at-risk mental states may be informative. Third, the subsampling between nonremitters and converters presented a challenge. Because of the limited sample sizes, we chose to use 2 analysis subsets, comparing healthy controls with remitters and nonremitters as well as healthy controls with nonconverters and converters. It would be ideal to classify samples as healthy controls, remitters, nonremitters, and converters, which is a necessary consideration for subsequent studies with larger sample sizes. Finally, following up participants at UHR for psychosis for only 24 months, although informative, limited the definition of remitters, nonremitters, and nonconverters. Cognitive dedifferentiation phenomena in nonremitters suggest the likelihood of long-lasting cognitive changes, but a longer prospective study would help clarify the degree to which these changes are detrimental to other aspects of clinical outcomes. Such a study would validate remission status (eg, if these cases slip back to being UHR for psychosis) and elucidate potential biological agent underpinnings responsible for the deficit.

## Conclusions

To our knowledge, to date, this study had 1 of the largest single-site samples of individuals at UHR for psychosis. It replicates findings in the literature that cognition is impaired before the onset of psychosis. Baseline cognitive impairment differentiates nonremitters with more enduring symptomatology from healthy controls and individuals at UHR for psychosis whose UHR status later remits. Although predominantly a trait, cognitive architecture shows subtle changes over time in nonremitting individuals at UHR for psychosis. These cognitive architecture changes are associated with functional outcomes and may herald a conversion to psychosis and a cognitive architecture similar to schizophrenia.

## Notes

Supplement. **eAppendix 1.** Statistical Approaches for Testing Principal Component Loadings: Data Analysis to Test Cognitive Structure Differences Across Groups Prospectively **eAppendix 2.** Concept of Differentiation and De-Differentiation of Cognitive Factors Tested via Changes in Factor Loadings: Kolmogorov-Smirnov Test for Approximating De-Differentiation **eAppendix 3.** Data Analysis Workflow: Discussion of Data-Analytic Strategies Carried Out for the Current Report **eTable 1.** Ordinal Regression and Post-Hoc Tests for Baseline Cognitive Prediction of Group Membership **eTable 2.** Linear Mixed Models Elements for Cognitive Tests **eTable 3.** Linear Mixed Models Elements for Maturational Stage Investigation **eTable 4.** Linear Mixed Model Elements for UHR Ascertainment Age and Age-Related Trajectories **eTable 5.** Post-Hoc Linear Mixed Model for UHR Ascertainment Age and Age-Related Trajectories **eTable 6.** Marginal Homogeneity Test for Effect Distribution at Baseline and Follow-Up **eTable 7.** Kolmogorov-Smirnov Tests for Component Loading Comparisons **eTable 8.** Univariate One-Way ANOVA for Cognitive Components, by Group Comparisons **eTable 9.** Repeated Measures ANOVA **eFigure 1.** Distribution of Factor Loadings Across Groups at Baseline and Follow-Up **eFigure 2.** Baseline Cognitive Profiles by Group **eFigure 3.** BACS Digit Sequencing: Linear Mixed Model SPSS Path Plots by Healthy Controls, Non-Converters, Converters, Maturational Stage and Time **eFigure 4.** BACS Semantic Fluency (F): Linear Mixed Model SPSS Path Plots by Healthy Controls, Non-converters, Converters, Maturational Stage and Time **eFigure 5.** BACS Symbol Coding: Linear Mixed Model SPSS Path Plots by Healthy Controls, Non-Converters, Converters, Maturational Stage and Time **eFigure 6.** BACS Verbal Memory: Linear Mixed Model SPSS Path Plots by Healthy Controls, Remitters, Non-Remitters, Maturational Stage and Time **eFigure 7.** BACS Semantic Fluency (F): Linear Mixed Model SPSS Path Plots by Healthy Controls, Remitters, Non-Remitters, Maturational Stage and Time **eFigure 8.** Snakes in Grass (Reaction Time): Linear Mixed Model SPSS Path Plots of Predicted Score for Age Dependent Trajectories Across Groups **eFigure 9.** Snakes in Grass (Accuracy): Linear Mixed Model SPSS Path Plots of Predicted Score for Age Dependent Trajectories Across Groups
