# Supplementary material for: Longitudinal Cognitive Changes in Young Individuals at Ultrahigh Risk for Psychosis
Source: JAMA Psychiatry. 2018 Jul 25;75(9):929–39. doi: 10.1001/jamapsychiatry.2018.1668 (PMC6142925; doi:10.1001/jamapsychiatry.2018.1668)
Supplement: Supplement. — eAppendix 1. Statistical Approaches for Testing Principal Component Loadings: Data Analysis to Test Cognitive Structure Differences Across Groups Prospectively eAppendix 2. Concept of Differentiation and De-Differentiation of Cognitive Factors Tested via Changes in Factor Loadings: Kolmogorov-Smirnov Test for Approximating De-Differentiation eAppendix 3. Data Analysis Workflow: Discussion of Data-Analytic Strategies Carried Out for the Current Report eTable 1. Ordinal Regression and Post-Hoc Tests for Baseline Cognitive Prediction of Group Membership eTable 2. Linear Mixed Models Elements for Cognitive Tests eTable 3. Linear Mixed Models Elements for Maturational Stage Investigation eTable 4. Linear Mixed Model Elements for UHR Ascertainment Age and Age-Related Trajectories eTable 5. Post-Hoc Linear Mixed Model for UHR Ascertainment Age and Age-Related Trajectories eTable 6. Marginal Homogeneity Test for Effect Distribution at Baseline and Follow-Up eTable 7. Kolmogorov-Smirnov Tests for Component Loading Comparisons eTable 8. Univariate One-Way ANOVA for Cognitive Components, by Group Comparisons eTable 9. Repeated Measures ANOVA eFigure 1. Distribution of Factor Loadings Across Groups at Baseline and Follow-Up eFigure 2. Baseline Cognitive Profiles by Group eFigure 3. BACS Digit Sequencing: Linear Mixed Model SPSS Path Plots by Healthy Controls, Non-Converters, Converters, Maturational Stage and Time eFigure 4. BACS Semantic Fluency (F): Linear Mixed Model SPSS Path Plots by Healthy Controls, Non-converters, Converters, Maturational Stage and Time eFigure 5. BACS Symbol Coding: Linear Mixed Model SPSS Path Plots by Healthy Controls, Non-Converters, Converters, Maturational Stage and Time eFigure 6. BACS Verbal Memory: Linear Mixed Model SPSS Path Plots by Healthy Controls, Remitters, Non-Remitters, Maturational Stage and Time eFigure 7. BACS Semantic Fluency (F): Linear Mixed Model SPSS Path Plots by Healthy Controls, Remitters, Non-Remitters, Maturational Stage a [file jamapsychiatry-e181668-s001.pdf]

Supplementary Online Content

Lam M, Lee J, Rapisarda A, et al. Longitudinal cognitive changes in young individuals at ultrahigh risk for psychosis. *JAMA Psychiatry*. Published online July 25, 2018. doi:10.1001/jamapsychiatry.2018.1668

eAppendix 1. Statistical Approaches for Testing Principal Component Loadings: Data Analysis to Test Cognitive Structure Differences Across Groups Prospectively

eAppendix 2. Concept of Differentiation and De-Differentiation of Cognitive Factors Tested via Changes in Factor Loadings: Kolmogorov-Smirnov Test for Approximating De-Differentiation

eAppendix 3. Data Analysis Workflow: Discussion of Data-Analytic Strategies Carried Out for the Current Report

eTable 1. Ordinal Regression and Post-Hoc Tests for Baseline Cognitive Prediction of Group Membership

eTable 2. Linear Mixed Models Elements for Cognitive Tests

eTable 3. Linear Mixed Models Elements for Maturational Stage Investigation

eTable 4. Linear Mixed Model Elements for UHR Ascertainment Age and Age-Related Trajectories

eTable 5. Post-Hoc Linear Mixed Model for UHR Ascertainment Age and Age-Related Trajectories

eTable 6. Marginal Homogeneity Test for Effect Distribution at Baseline and Follow-Up

eTable 7. Kolmogorov-Smirnov Tests for Component Loading Comparisons

eTable 8. Univariate One-Way ANOVA for Cognitive Components, by Group Comparisons

eTable 9. Repeated Measures ANOVA

eFigure 1. Distribution of Factor Loadings Across Groups at Baseline and Follow-Up

eFigure 2. Baseline Cognitive Profiles by Group

eFigure 3. BACS Digit Sequencing: Linear Mixed Model SPSS Path Plots by Healthy Controls, Non-Converters, Converters, Maturational Stage and Time

eFigure 4. BACS Semantic Fluency (F): Linear Mixed Model SPSS Path Plots by Healthy Controls, Non-converters, Converters, Maturational Stage and Time

© 2018 Lam L et al. *JAMA Psychiatry.*

eFigure 5. BACS Symbol Coding: Linear Mixed Model SPSS Path Plots by Healthy Controls, Non-Converters, Converters, Maturational Stage and Time

eFigure 6. BACS Verbal Memory: Linear Mixed Model SPSS Path Plots by Healthy Controls, Remitters, Non-Remitters, Maturational Stage and Time

eFigure 7. BACS Semantic Fluency (F): Linear Mixed Model SPSS Path Plots by Healthy Controls, Remitters, Non-Remitters, Maturational Stage and Time

eFigure 8. Snakes in Grass (Reaction Time): Linear Mixed Model SPSS Path Plots of Predicted Score for Age Dependent Trajectories Across Groups

eFigure 9. Snakes in Grass (Accuracy): Linear Mixed Model SPSS Path Plots of Predicted Score for Age Dependent Trajectories Across Groups

This supplementary material has been provided by the authors to give readers additional information about their work.

© 2018 Lam L et al. *JAMA Psychiatry.*

eAppendix 1. Statistical Approaches for Testing Principal Component Loadings: Data Analysis to Test Cognitive Structure Differences Across Groups Prospectively

Principal Component Analysis (PCA) has been used as a technique for data reduction and to identify differences in data structure. It is proposed that PCA and its loadings could potentially be utilized as a means of assessing differences in data structure for cognition between groups of subjects and within subjects over time. There have been various methods to compare PCA loadings e.g. Tucker Congruence Coefficient (TCC; Lorenzo-Seva & Ten Berge, 2006) and Correlation Coefficient Comparison algorithms [2].

The TCC had been designed to measure equivalence of latent trait via EFA (Tucker, 1951). However, there are limitations to the methodology [4,5]. Some of the issues that were highlighted with the use of the TCC were i) the lack of hypothesis testing - though Lorenzo-seva and Ten Berge (2006) suggested that R_{TCC} = .85-.95 indicates congruent factor structure, it is notable that these were obtained from consensus ratings and not necessary statistical in nature [4] ii) the TCC was developed mainly for inter sample comparisons, and hence the notion of repeated testing within a sample would tend to inflate TCC values iii) TCC is a similarity index hence if the hypothesis is one that suggest that factors are differential the TCC would not have been an optimal test to show difference iv) factor similarity is not synonymous with factor invariance. To highlight this point, two 10-item vector s1 = {.872, .736, .622, .502, .213, .123, .021, .014, .003, .002} and s2 = {.523, .441, .373, .301, .128, .074, .013, .008, .001, .001} is likely to give a very high congruent rating close to if not beyond .90. The reason being the loadings in vector s2 is in fact a function of s1 i.e. f(s2) = 0.6(s1) with a coefficient of a constant 0.6. In this circumstance, the factor loadings might be invariant, but not similar.

The second approach of involve using various correlation estimators [6]. While the approach is compelling, and allows between and within subject comparisons, considering sample sizes and repeated sampling, the approach involves singular coefficient comparisons. The challenge with performing singular comparisons results in massive multiple testing, reducing statistical power and introducing noise to the interpretation of coefficient differences. The challenge is escalated when factor loadings of large number of overlapping items from a neuropsychological test battery is compared.

© 2018 Lam L et al. *JAMA Psychiatry.*

eAppendix 2. Concept of Differentiation and De-Differentiation of Cognitive Factors Tested via Changes in Factor Loadings: Kolmogorov-Smirnov Test for Approximating De-Differentiation

The following discussion is restricted to orthogonal PCA. PCA oblique rotations are not chosen, as it is challenging to control for *theta* Θ (angle between principal component vectors) satisfactorily in higher dimension solutions. Moreover, in component comparisons, the objective of testing the data structure is not to minimize cross loadings across components, which is often a reason oblique rotations are considered. Rather, to test the dedifferentiation hypothesis, covered in the main text, one should be looking for items that cross load in the context of orthogonal PCA. Component loadings from PCA follow a gamma distribution (see eFigure 1). To compare component similarity as a function of component load, a non-parametric methodology is employed using the Kolmogorov-Smirnov (KS) test comparisons of PCA solutions.

Kolmogorov-Smirnov Test

The KS test brings significant advantages to the test of number sequences without consideration of the distribution function underlying the number sequences. In this case, the number sequences tested are the PCA loadings. The KS test makes use of the Cumulative Distribution Function (CDF). Specifically, the test establishes if continuous numbers on two vectors have the same distribution. The hypothesis test comes from the largest deviation of the CDF of both vectors, where the largest absolute difference D_{max} can be calculated. Hence the statistic of the KS Test is given as follows:

$$D_n = \max_x | F_n^1 (x_1) - F_n^2 (x_2)|$$

Where, $D_n = D_{max}$, F^1 = vector 1, F^2 = vector 2, *n* = number of items in vectors, *x* = distributional function with which vectors were drawn from. The logic follows that if the vectors were the same or very similar, then, $\lim_{n\to\infty} D_n = 0$. The converse is true, of the vectors were different and that they were drawn from totally different distributions where $x_1 \neq x_2$ then, $\lim_{n\to\infty} D_n \approx 1$. It is argued that this approach of testing PCA loadings would be sensitive to the overall perturbations in factor loadings. The test strategy of examining between group factor loadings is to perform KS-test on each of the five factors obtained in the prior PCA analysis of the cognitive test battery performed in each of the groups using controls as the reference distribution.

© 2018 Lam L et al. *JAMA Psychiatry.*

eAppendix 3. Data Analysis Workflow: Discussion of Data-Analytic Strategies Carried Out for the Current Report

The following data analysis workflow chart details the data analysis workflow that was carried out as part of the investigation reported in the current manuscript. Prior to data analysis basic preprocessing of the cognitive data was undertaken. Demographical factors such as age and gender were adjusted for using baseline age and gender via the following linear regression model:

$$Test(n) = \beta Age + \beta Gender + \beta Age^2 + \beta Age * Gender + \beta Age^2 * Gender + \beta zResidual$$

Where, Test(n) represents the vector of cognitive tests administered to all participants in the study. Linear and non-linear effects of age at baseline were adjusted for to ensure latent differences that are brought about by Age at baseline did not result in extraneous effects during inter-group comparisons downstream. zResiduals represents the adjusted standardized scores after adjustment for baseline demographic factors. Special note should be taken that only baseline age and gender are used for adjustment of cognitive performance for each of the time points. The rationale for doing so is two fold- First, neurodevelopmental trajectories related to cognition may be embedded within the trajectories associated with clinical outcomes. Hence, we adopted this strategy of demographic adjustment rather than using Age within subsequent longitudinal modelling, of which adjusting away the Age*Time effect could remove potentially interesting differential maturational effects across clinical groups. Second, even though there does not appear to be significant differences in terms of age at recruitment of either healthy controls or UHR individuals, one cannot assume that cognition and age correlations do not exist. Hence, the approach that we employed is most likely to balance methodological rigor for the analysis of cognitive data, but at the same time giving enough room for longitudinal effects to vary.

Ordinal logistic regression was conducted on (i) healthy controls, at-risk individuals and converters; and (ii) healthy controls, remitters, and non-remitters. These analyses aimed to establish baseline differences between groups. Each cognitive test was entered as a predictor to group membership in multiple univariate models. Test of parallel lines across all cognitive tests were not significant, which indicate that the method was interpretable for the purpose of the current report. At the preliminary stage of the analysis, a liberal approach allowed significant thresholds of $p < 0.05$ to select cognitive tests for subsequent analysis. This is however, already considered stricter than recommended p-values of between 0.1-0.15 [7]. Ordinal Logistic Regression was implemented via the PLUM module in IBM SPSS 22.0

Prospective modeling of cognitive change was completed via linear mixed models. Time points were coded in 0.5 year increments with baseline as “0”. Intercept and Time were modeled as random effects, using unstructured covariance structure. Time, Time2, Group, Group*Time, Group*Time2 were modeled as fixed effects. Test performance was modeled as dependent variables in separate models. Two sets of Linear Mixed Model analysis were conducted for (i) Healthy Controls, Remitters, and Non-Remitters, and (ii) Healthy Controls, Non-Converters, and Converters. The overall model was:

$$Test(n) = \beta 0 + \beta 1 * Time + \beta 2 * Group + \beta 3 * Time^2 + \beta 4 * Group * Time + \beta 5 * Group * Time^2 \quad \text{- (1)}$$

A liberal p-value of .05 was also used to identify potential group by time interaction effects. Additionally, to investigate the possibility of differential maturational trajectories in our sample, a median plot was carried out for the Age variable at baseline, resulting in Age <= 21 and Age >= 22 groups based on the baseline demographics. The Linear Mixed Model analysis then repeated for both sets of analysis via the following model:

© 2018 Lam L et al. *JAMA Psychiatry.*

$$Test(n) = \beta 0 + \beta 1 * Time + \beta 2 * Group + \beta 3 Maturational\ Stage + \beta 4 * Time^2 + \beta 5 * Group * Time * Maturational\ Stage + \beta 6 * Group * Time^2 * Maturational\ Stage$$ - (2)

We also attempted modeling age continuously, including age at ascertainment of UHR status ("UHR Age"), along with actual age changes over time, as indicated in the following model:

$$Test(n) = \beta 0 + \beta 1 * Time + \beta 2 * Group + \beta 3 UHRAge + \beta 4 Age + \beta 4 * Time^2 + \beta 5 * UHRAge * Age * Time * Group + \beta 6 * UHRAge * Age * Time^2 * Group$$ – (3)

In models (1), (2) and (3), Test(n) represents the vector of all test included in the cognitive assessment battery administered to each participant. Post-hoc tests were then conducted to evaluate potential maturational stage*Time effects and Age dependent trajectory in each of the clinical groups. Linear Mixed Modelling was implemented via the MIXED module in IBM SPSS 22.0.

Distributions of predicted scores from linear mixed models across cognitive tests were compared between baseline and 24- month follow-up for each group (healthy controls, remitters, non-remitters, non-converters and converters) using the Stuart-Maxwell marginal homogeneity (MH) test implemented in IBM SPSS 22.0.

Principal components analysis of tests that were found significant (P < .05) in the initial ordinal regression analysis was carried out for healthy controls, remitters and non-remitters for both baseline (BL) and 24 month (FU) time points. Factor loading patterns were then compared using the Kolmogorov-Smirnov test via the ks.boot() module in R 3.4.1 [8], that allow bootstrapping of the input vectors. Default bootstrapping parameters of n = 1000 resampling option was selected.

© 2018 Lam L et al. *JAMA Psychiatry.*

Data Analysis Workflow

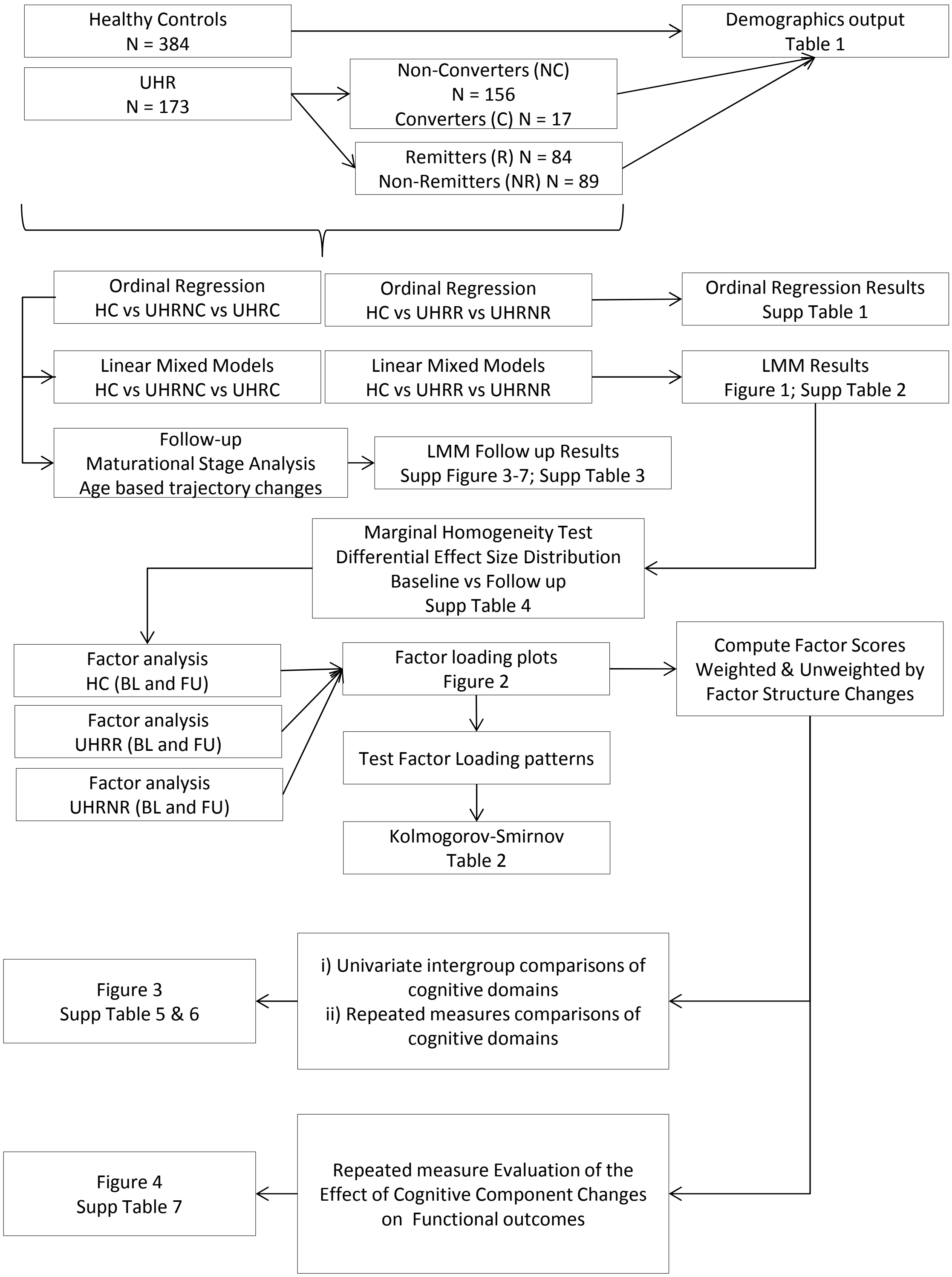

© 2018 Lam L et al. *JAMA Psychiatry.*

Factor scores weighted for differential factor structure perturbations were computed based on the following model:

$$Cognitive\ Domain\ Score = \sum_{i=1}^{n}[(PCA.loading_n * Subtest_n)] * L$$

Where n is the vector of tests included in the PCA. And $Subtest_n$ represents the vector of actual task performance of vector n. L represents the group vector of healthy controls, remitters and non-remitters. There are ten cognitive domain scores that reported, Social Cognition, Attention, Verbal, General Cognitive Function (GCF) and Perception for follow-up and baseline. These factor scores were standardized using healthy controls as the reference group. Non-weighted factor scores were computed similarly, however for non-weighted scores factor loadings for items belonging to a particular domain were constrained to 1 while items not belonging to the domain were constrained to 0. Cognitive domain change scores were computed by subtracting follow-up scores with baseline scores. Two sets of change scores were also computed i) weighted scores, representing the change score weighted by factor structure changes over time ii) non-weighted scores

Downstream comparisons of the factor scores were carried out using One-Way ANOVA with Bonferroni Correction for between subject differences and Repeated Measures ANOVA for 2 (BL vs FU) x Group (HC v UHRR v UHRNR) for Time based comparisons. Further examination on how changes in component scores between baseline and follow-up might account for social and occupational functioning outcomes. The SOFAS differential represented the social and occupational functioning range within 6 months of the assessment time point. The larger the range the less likely an individual was doing well. Multivariate repeated measures general linear model was carried out on the percentage SOFAS differential at baseline and follow up. Additional a time*group term was included to assess differential changes of functioning across healthy controls, remitters and non-remitters and additional cognitive component change* time elements were included to examine if changes in cognition might explain differential functional changes across groups. All cognitive component change scores were included in the multivariate model. Analyses were carried out using the GLM module in IBM SPSS 22.0.

© 2018 Lam L et al. *JAMA Psychiatry.*

**eTable 1.** Ordinal Regression and Post-Hoc tests for Baseline Cognitive Prediction of Group Membership								
	OR (95% CI)		Post-Hoc Independent T-tests					
	HC,UHRNC, UHRC	HC,UHRR,UHRNR	HC UHRNC	HC UHRC	UHRNC UHRC	HC UHRR	HC UHRNR	UHRR UHRNR
BACS Verbal Memory	1.534 (1.277-1.842)**	1.587 (1.324-1.901)**	4.086**	3.010*	1.237	1.920	5.514**	2.639*
BACS Digit Sequencing	1.526 (1.272-1.831)**	1.532 (1.281-1.83)**	4.040**	3.083*	1.282	2.525*	4.757**	1.564
BACS Token Motor Task	2.007 (1.632-2.467)**	1.960 (1.604-2.396)**	5.872**	4.398**	2.489*	4.024**	6.151**	1.903
BACS Semantic Fluency (total)	1.457 (1.222-1.738)**	1.436 (1.209-1.707)**	2.888*	4.651**	2.860*	1.880	4.32**	1.645
BACS Symbol Coding	2.045 (1.685-2.483)**	1.982 (1.644-2.389)**	6.525**	5.281**	2.450*	3.998**	7.614**	2.581*
BACS Tower of London	1.338 (1.116-1.604)*	1.352 (1.13-1.618)*	2.248*	3.223*	2.058*	.916	3.675**	2.029*
WMSIIIss	1.831 (1.501-2.235)**	1.902 (1.562-2.316)**	5.577**	3.457*	1.383	3.16*	6.326**	2.757*
HISOC Affect/Expression	2.207 (1.719-2.833)**	2.216 (1.73-2.838)**	5.231**	5.174**	2.203*	3.029*	7.046**	2.86*
HISOC Behavior/Language	2.151 (1.66-2.789)**	2.229 (1.719-2.889)**	5.092**	4.336**	1.686	3.133*	6.171**	2.411*
HISOC Comm/Social	1.701 (1.339-2.161)**	1.784 (1.404-2.267)**	3.250*	4.135**	2.124*	.860	5.996**	3.809**
SGTargetrt	1.338 (1.113-1.608)*	1.303 (1.089-1.559)*	2.504*	2.445*	1.356	1.208	3.026*	1.346
SGDistractrt	1.316 (1.1-1.575)*	1.302 (1.092-1.554)*	2.749*	1.512	.416	1.604	2.792*	.883
CPT2d	1.325 (1.11-1.582)*	1.3 (1.092-1.547)*	2.159*	3.285*	2.089*	2.423*	2.168*	-.079
CPT3d	1.295 (1.084-1.548)*	1.309 (1.101-1.558)*	1.685	3.701**	2.632*	.940	3.423*	1.735
CPT4d	1.523 (1.257-1.844)**	1.492 (1.238-1.799)**	3.839**	2.389*	.965	3.301*	3.305*	.075
* p < .05; ** p < .001								
Note: BACS: Brief Assessment of Cognition in Schizophrenia; HISOC: High Risk Social Skills; BDIT: Binocular Depth Inversion; WMSSS: Wechsler Memory Scale - Spatial Span; SG: Snakes in the Grass; BABBLE: Babble Task; CPT: Continuous Performance Task								
HC: Healthy Controls; UHRNC: Non-Converters; UHRC: Converters; UHRR: Remitters; UHRNR:Non-Remitters								

© 2018 Lam L et al. *JAMA Psychiatry.*

**eTable 2.** Linear Mixed Models Elements for Cognitive Tests						
	β_0	β(Time)	β(Time^2)	β(Group)	β(Group*Time)	β(Group*Time^2)
Healthy Controls, Non-converters, and Converters						
BACS Verbal Memory	-0.016(0.05)	**0.321(0.129)***	-0.033(0.063)	**-0.417(0.08)****	0.197(0.168)	-0.072(0.082)
BACS Digit Sequencing	-0.014(0.053)	**0.321(0.121)***	-0.086(0.059)	**-0.378(0.084)****	-0.063(0.157)	0.067(0.076)
BACS Token Motor Task	-0.01(0.048)	**0.426(0.127)***	**-0.175(0.062)***	**-0.549(0.077)****	0.06(0.165)	-0.01(0.08)
BACS Semantic Fluency (animals)	0.015(0.052)	0.006(0.114)	-0.004(0.056)	**-0.345(0.083)****	0.14(0.148)	-0.026(0.072)
BACS Semantic Fluency (fruits)	0.004(0.053)	-0.151(0.131)	0.111(0.064)	**-0.275(0.083)***	0.147(0.169)	-0.1(0.083)
BACS Semantic Fluency (veg)	0.014(0.053)	**-0.378(0.119)***	**0.186(0.058)***	**-0.322(0.085)****	**0.37(0.154)***	-0.14(0.075)
BACS Semantic Fluency (total)	0.014(0.054)	-0.183(0.096)	**0.103(0.047)***	**-0.397(0.086)****	0.231(0.124)	-0.088(0.06)
BACS Symbol Coding	-0.007(0.053)	**0.246(0.101)***	-0.04(0.049)	**-0.646(0.084)****	**0.262(0.131)***	-0.105(0.063)
BACS Tower of London	-0.003(0.049)	**0.504(0.142)****	**-0.201(0.07)***	**-0.298(0.077)****	-0.126(0.187)	0.121(0.091)
HISOC Affect/Expression	-0.014(0.057)	**-0.43(0.152)***	0.142(0.075)	**-0.695(0.097)****	0.327(0.203)	-0.155(0.099)
HISOC Behavior/Language	-0.007(0.057)	0.051(0.169)	-0.07(0.083)	**-0.625(0.096)****	0.284(0.23)	-0.135(0.112)
HISOC Comm/Social	-0.002(0.057)	-0.145(0.154)	0.005(0.076)	**-0.492(0.097)****	0.079(0.208)	-0.014(0.102)
HISOC Average	-0.009(0.057)	-0.209(0.151)	0.035(0.074)	**-0.684(0.096)****	0.27(0.203)	-0.116(0.099)
* $p < 0.05$; ** $p < 0.001$						
Note: BACS: Brief Assessment of Cognition in Schizophrenia; HISOC: High Risk Social Skills; BDIT: Binocular Depth Inversion; WMSSS Wechsler Memory Scale - Spatial Span; SG: Snakes in the Grass; BABBLE: Babble Task; CPT: Continuous Performance Task						
β0: Intercept; β(Time): Effect of Time; β(Time2); Non-linear Effect of Time; β(Group*Time): Interaction/Differential Group Effects over time						
β(Group*Time2): Non-linear Interaction/Differential Group Effects over time						

© 2018 Lam L et al. *JAMA Psychiatry.*

eTable 2 cont'd. Linear Mixed Models Elements for Cognitive Tests						
	β_0	β(Time)	β(Time^2)	β(Group)	β(Group*Time)	β(Group*Time^2)
Healthy Controls, Non-converters, and Converters						
BDIT Total	0.007(0.053)	-0.14(0.106)	0.046(0.051)	**-0.166(0.084)***	-0.18(0.138)	0.081(0.066)
BDIT Total Index	0.007(0.053)	-0.124(0.106)	0.04(0.052)	-0.164(0.084)	-0.192(0.138)	0.085(0.066)
BDIT Faces	0.014(0.053)	-0.037(0.112)	0.002(0.054)	-0.145(0.085)	-0.265(0.145)	0.127(0.07)
BDIT Faces Index	0.014(0.053)	-0.033(0.111)	0(0.054)	-0.146(0.085)	-0.27(0.145)	0.129(0.07)
WMSIIIss	-0.009(0.049)	-0.123(0.124)	0.118(0.061)	**-0.472(0.078)****	0.289(0.16)	-0.13(0.078)
SGTargetrt	-0.002(0.05)	**0.593(0.124)****	**-0.19(0.061)***	**-0.25(0.08)***	0.256(0.161)	-0.066(0.078)
SGTargetAcc	0.008(0.052)	0.187(0.136)	-0.059(0.067)	**-0.219(0.082)***	0.18(0.178)	-0.084(0.086)
SGDistractrt	-0.009(0.052)	**0.597(0.13)****	**-0.175(0.063)***	**-0.242(0.083)***	0.27(0.169)	-0.083(0.082)
SGDistractAcc	0.002(0.05)	-0.042(0.136)	**0.066(0.067)***	**-0.176(0.08)***	0.359(0.179)	-0.148(0.087)
SGAllsnakesrt	0.001(0.052)	**0.606(0.15)****	**-0.254(0.073)***	-0.149(0.083)	-0.052(0.197)	0.066(0.096)
SGAllsnakesacc	0.001(0.052)	**0.606(0.15)****	**-0.254(0.073)***	-0.149(0.083)	-0.052(0.197)	0.066(0.096)
BABBLEphrase	0.004(0.051)	0.214(0.136)	-0.078(0.067)	-0.035(0.081)	-0.044(0.179)	0.037(0.087)
BABBLEwords	0.001(0.052)	**0.443(0.113)****	**-0.157(0.055)***	-0.024(0.084)	0.051(0.147)	0.021(0.071)
CPT2d	0.005(0.052)	0.072(0.153)	-0.005(0.075)	**-0.3(0.082)****	0.317(0.2)	-0.178(0.098)
CPT3d	0.016(0.053)	0.007(0.135)	0.05(0.066)	**-0.275(0.084)***	0.29(0.175)	-0.139(0.085)
CPT4d	-0.001(0.05)	0.079(0.116)	0.006(0.057)	**-0.346(0.079)****	**0.44(0.15)***	**-0.162(0.074)***
CPTaverage	0.005(0.051)	0.082(0.11)	0.013(0.054)	**-0.369(0.081)****	**0.367(0.142)***	**-0.161(0.069)***
* $p < 0.05$; ** $p < 0.001$						
Note: BACS: Brief Assessment of Cognition in Schizophrenia; HISOC: High Risk Social Skills; BDIT: Binocular Depth Inversion; WMSSS Wechsler Memory Scale - Spatial Span; SG: Snakes in the Grass; BABBLE: Babble Task; CPT: Continuous Performance Task						
β0: Intercept; β(Time): Effect of Time; β(Time2); Non-linear Effect of Time; β(Group*Time): Interaction/Differential Group Effects over time						
β(Group*Time2): Non-linear Interaction/Differential Group Effects over time						

© 2018 Lam L et al. *JAMA Psychiatry.*

eTable 2 cont'd. Linear Mixed Models Elements for Cognitive Tests						
	β_0	β(Time)	β(Time^2)	β(Group)	β(Group*Time)	β(Group*Time^2)
Healthy Controls, Remitters, and Non-Remitters						
BACS Verbal Memory	-0.005(-0.103)	0.23(-0.013)	0.009(-0.111)	**-0.339(-0.45)****	**0.274(0.042)***	-0.108(-0.223)
BACS Digit Sequencing	-0.012(-0.116)	**0.241(0.012)***	-0.047(-0.159)	**-0.287(-0.405)****	0.049(-0.168)	0.005(-0.102)
BACS Token Motor Task	-0.019(-0.114)	**0.345(0.106)***	**-0.137(-0.254)***	**-0.384(-0.492)****	0.138(-0.09)	-0.049(-0.161)
BACS Semantic Fluency (animals)	0.011(-0.091)	-0.007(-0.223)	0.003(-0.103)	**-0.243(-0.361)****	0.108(-0.097)	-0.02(-0.121)
BACS Semantic Fluency (fruits)	-0.009(-0.112)	-0.171(-0.417)	0.118(-0.003)	**-0.179(-0.297)***	0.133(-0.101)	-0.081(-0.196)
BACS Semantic Fluency (veg)	-0.004(-0.108)	**-0.34(-0.564)***	**0.167(0.058)***	**-0.202(-0.322)***	**0.23(0.017)***	-0.079(-0.184)
BACS Semantic Fluency (total)	0(-0.105)	-0.173(-0.354)	**0.096(0.008)***	**-0.264(-0.385)****	0.156(-0.015)	-0.053(-0.137)
BACS Symbol Coding	-0.011(-0.113)	**0.283(0.092)***	-0.059(-0.153)	**-0.468(-0.586)****	0.131(-0.051)	-0.047(-0.136)
BACS Tower of London	-0.006(-0.101)	**0.44(0.172)***	**-0.169(-0.301)***	**-0.213(-0.322)****	-0.023(-0.281)	0.056(-0.072)
HISOC Affect/Expression	-0.011(-0.123)	-0.395(-0.686)	**0.123(-0.02)***	-0.551(-0.697)	0.211(-0.091)	-0.096(-0.245)
HISOC Behavior/Language	-0.003(-0.114)	0.063(-0.262)	-0.079(-0.239)	**-0.499(-0.645)****	0.211(-0.13)	-0.094(-0.262)
HISOC Comm/Social	0.012(-0.1)	-0.156(-0.452)	0.005(-0.141)	**-0.422(-0.568)****	0.091(-0.217)	-0.009(-0.162)
HISOC Average	-0.003(-0.114)	-0.189(-0.479)	0.021(-0.122)	**-0.552(-0.697)****	0.189(-0.112)	-0.071(-0.219)
* p < 0.05; ** p < 0.001						
Note: BACS: Brief Assessment of Cognition in Schizophrenia; HISOC: High Risk Social Skills; BDIT: Binocular Depth Inversion; WMSSS Wechsler Memory Scale - Spatial Span; SG: Snakes in the Grass; BABBLE: Babble Task; CPT: Continuous Performance Task						
β0: Intercept; β(Time): Effect of Time; β(Time2); Non-linear Effect of Time; β(Group*Time): Interaction/Differential Group Effects over time						
β(Group*Time2): Non-linear Interaction/Differential Group Effects over time						

© 2018 Lam L et al. *JAMA Psychiatry.*

eTable 2 cont'd. Linear Mixed Models Elements for Cognitive Tests						
	β_0	β(Time)	β(Time^2)	β(Group)	β(Group*Time)	β(Group*Time^2)
Healthy Controls, Remitters, and Non-Remitters						
BDIT Total	0.008(-0.096)	-0.17(-0.372)	0.061(-0.037)	-0.112(-0.231)	-0.117(-0.309)	0.048(-0.045)
BDIT Total Index	0.008(-0.096)	-0.158(-0.36)	0.057(-0.04)	-0.112(-0.231)	-0.12(-0.312)	0.048(-0.045)
BDIT Faces	0.006(-0.099)	-0.075(-0.288)	0.022(-0.081)	-0.076(-0.195)	-0.18(-0.383)	0.081(-0.018)
BDIT Faces Index	0.006(-0.099)	-0.07(-0.283)	0.02(-0.084)	-0.076(-0.196)	-0.184(-0.386)	0.083(-0.015)
WMSIIIss	-0.005(-0.1)	-0.111(-0.344)	0.11(-0.005)	**-0.359(-0.468)****	0.196(-0.025)	-0.084(-0.193)
SGTargetrt	-0.005(-0.104)	**0.555(0.322)****	**-0.167(-0.281)***	**-0.165(-0.279)***	**0.219(-0.004)***	-0.077(-0.186)
SGTargetAcc	-0.013(-0.115)	0.253(-0.005)	-0.091(-0.217)	**-0.126(-0.243)***	0.048(-0.199)	-0.02(-0.141)
SGDistractrt	-0.011(-0.112)	**0.57(0.325)****	**-0.158(-0.277)***	**-0.167(-0.283)***	0.226(-0.009)	-0.085(-0.199)
SGDistractAcc	-0.009(-0.107)	0.081(-0.177)	0.002(-0.123)	-0.109(-0.222)	0.097(-0.15)	-0.018(-0.139)
SGAllsnakesrt	-0.014(-0.117)	**0.644(0.36)****	**-0.27(-0.409)****	-0.083(-0.201)	-0.101(-0.374)	0.077(-0.057)
SGAllsnakesacc	-0.014(-0.117)	**0.644(0.36)****	**-0.27(-0.409)****	-0.083(-0.201)	-0.101(-0.374)	0.077(-0.057)
BABBLEphrase	0.021(-0.078)	**0.277(0.02)***	-0.112(-0.238)	-0.061(-0.175)	-0.105(-0.352)	0.068(-0.053)
BABBLEwords	0.014(-0.087)	**0.512(0.299)****	**-0.192(-0.297)****	-0.044(-0.161)	-0.049(-0.251)	0.062(-0.037)
CPT2d	-0.019(-0.121)	0.159(-0.13)	-0.048(-0.19)	**-0.17(-0.286)***	0.114(-0.163)	-0.072(-0.209)
CPT3d	0.009(-0.095)	0.072(-0.182)	0.011(-0.113)	**-0.199(-0.319)***	0.135(-0.108)	-0.048(-0.167)
CPT4d	-0.014(-0.111)	0.199(-0.021)	-0.052(-0.161)	**-0.233(-0.344)***	0.177(-0.032)	-0.046(-0.149)
CPTaverage	-0.008(-0.108)	0.188(-0.021)	-0.041(-0.144)	**-0.251(-0.366)***	0.142(-0.056)	-0.048(-0.146)
* p < 0.05; ** p < 0.001						
Note: BACS: Brief Assessment of Cognition in Schizophrenia; HISOC: High Risk Social Skills; BDIT: Binocular Depth Inversion; WMSSS Wechsler Memory Scale - Spatial Span; SG: Snakes in the Grass; BABBLE: Babble Task; CPT: Continuous Performance Task						
β0: Intercept; β(Time): Effect of Time; β(Time2); Non-linear Effect of Time; β(Group*Time): Interaction/Differential Group Effects over time						
β(Group*Time2): Non-linear Interaction/Differential Group Effects over time						

© 2018 Lam L et al. *JAMA Psychiatry.*

**eTable 3.** Linear Mixed Models Elements for Maturational Stage investigation						
	β_0	β(Time)	β(Time^2)	β(MatStage)	β(Group*Time *MatStage)	β(Group*Time^2 *MatStage)
Healthy Controls, Non-converters, and Converters						
BACS Verbal Memory	-0.029 (0.067)	**0.361 (0.102)****	-0.052 (0.05)	0.013 (0.078)	0.291 (0.201)	-0.086 (0.1)
BACS Digit Sequencing	-0.014 (0.07)	**0.27 (0.087)***	-0.062 (0.042)	0.008 (0.083)	**0.294 (0.125)***	0.058 (0.032)
BACS Token Motor Task	-0.019 (0.063)	**0.526 (0.1)****	**-0.216 (0.049)****	-0.023 (0.072)	-0.329 (0.195)	0.144 (0.098)
BACS Semantic Fluency (animals)	0.049 (0.07)	0.022 (0.09)	-0.004 (0.044)	-0.088 (0.083)	0.227 (0.179)	-0.066 (0.088)
BACS Semantic Fluency (fruits)	0.054 (0.069)	-0.148 (0.103)	**0.105 (0.05)***	-0.077 (0.081)	0.358 (0.202)	**-0.209 (0.1)***
BACS Semantic Fluency (veg)	-0.007 (0.071)	**-0.192 (0.094)***	**0.104 (0.046)***	-0.019 (0.083)	0.041 (0.185)	0.012 (0.092)
BACS Semantic Fluency (total)	0.045 (0.073)	-0.123 (0.075)	**0.078 (0.037)***	-0.08 (0.087)	0.271 (0.151)	-0.11 (0.074)
BACS Symbol Coding	0.018 (0.072)	**0.31 (0.08)****	-0.068 (0.039)	-0.06 (0.087)	**0.335 (0.16)***	-0.127 (0.078)
BACS Tower of London	-0.04 (0.062)	**0.354 (0.113)***	**-0.113 (0.056)***	0.035 (0.068)	0.306 (0.218)	-0.115 (0.111)
HISOC Affect/Expression	-0.071 (0.076)	**-0.27 (0.125)***	0.064 (0.062)	0.082 (0.091)	0.011 (0.255)	0.005 (0.128)
HISOC Behavior/Language	-0.071 (0.074)	0.139 (0.14)	-0.115 (0.069)	0.108 (0.085)	0.259 (0.283)	-0.099 (0.143)
HISOC Comm/Social	-0.055 (0.074)	-0.116 (0.127)	-0.006 (0.063)	0.081 (0.086)	0.037 (0.258)	0.02 (0.13)
HISOC Average	-0.081 (0.075)	-0.101 (0.125)	-0.017 (0.062)	0.109 (0.089)	0.115 (0.254)	-0.029 (0.127)
* $p < 0.05$; ** $p < 0.001$						
Note: BACS: Brief Assessment of Cognition in Schizophrenia; HISOC: High Risk Social Skills; BDIT: Binocular Depth Inversion; WMSSS Wechsler Memory Scale - Spatial Span; SG: Snakes in the Grass; BABBLE: Babble Task; CPT: Continuous Performance Task						
MatStage: Maturational Stage - Median split of Age at baseline						
β0: Intercept; β(Time): Effect of Time; β(Time2); Non-linear Effect of Time; β(Group*Time*MatStage): Interaction/Differential Group & Maturational Effects over time						
β(Group*Time^2*MatStage): Non-linear Interaction/Differential Group & Maturational Effects Effects over time						

© 2018 Lam L et al. *JAMA Psychiatry.*

eTable 3 cont'd. Linear Mixed Models Elements for Maturational Stage investigation						
	β_0	β(Time)	β(Time^2)	β(MatStage)	β(Group*Time *MatStage)	β(Group*Time^2 *MatStage)
Healthy Controls, Non-converters, and Converters						
BDIT Total	0.02 (0.072)	**-0.218 (0.084)***	**0.085 (0.041)***	-0.014 (0.087)	-0.1 (0.17)	0.023 (0.082)
BDIT Total Index	0.017 (0.072)	**-0.205 (0.084)***	**0.08 (0.041)***	-0.007 (0.087)	-0.114 (0.17)	0.029 (0.082)
BDIT Faces	0.012 (0.072)	-0.125 (0.088)	0.046 (0.043)	0.016 (0.087)	-0.26 (0.177)	0.118 (0.086)
BDIT Faces Index	0.014 (0.072)	-0.124 (0.088)	0.045 (0.043)	0.013 (0.087)	-0.257 (0.177)	0.117 (0.086)
WMSIIIss	0.027 (0.065)	0.008 (0.098)	0.055 (0.048)	-0.089 (0.076)	0.117 (0.192)	-0.042 (0.096)
SGTargetrt	-0.012 (0.066)	**0.702 (0.098)****	**-0.228 (0.048)****	-0.048 (0.076)	0.066 (0.192)	-0.005 (0.095)
SGTargetAcc	0.078 (0.065)	**0.27 (0.107)***	-0.097 (0.053)	**-0.15 (0.071)***	0.062 (0.208)	-0.046 (0.105)
SGDistractrt	-0.021 (0.068)	**0.684 (0.103)****	**-0.203 (0.05)****	-0.034 (0.078)	0.196 (0.202)	-0.092 (0.1)
SGDistractAcc	0.035 (0.064)	0.144 (0.108)	-0.023 (0.053)	-0.097 (0.072)	0.025 (0.209)	0.037 (0.105)
SGAllsnakesrt	0.039 (0.066)	**0.521 (0.119)****	**-0.203 (0.058)****	-0.089 (0.074)	0.207 (0.229)	-0.078 (0.116)
SGAllsnakesacc	0.039 (0.066)	**0.521 (0.119)****	**-0.203 (0.058)****	-0.089 (0.074)	0.207 (0.229)	-0.078 (0.116)
BABBLEphrase	-0.001 (0.065)	0.2 (0.108)	-0.07 (0.053)	0.002 (0.072)	-0.056 (0.209)	0.052 (0.106)
BABBLEwords	0.009 (0.069)	**0.472 (0.089)****	**-0.168 (0.044)****	-0.033 (0.082)	-0.022 (0.177)	0.1 (0.087)
CPT2d	0.054 (0.067)	**0.257 (0.121)***	-0.107 (0.059)	-0.082 (0.077)	-0.017 (0.234)	0.04 (0.118)
CPT3d	-0.016 (0.069)	0.155 (0.106)	-0.023 (0.052)	0.035 (0.08)	0.045 (0.209)	-0.016 (0.104)
CPT4d	-0.026 (0.066)	**0.247 (0.092)***	-0.066 (0.045)	-0.014 (0.078)	0.279 (0.181)	-0.084 (0.09)
CPTaveraged	-0.016 (0.069)	**0.242 (0.087)***	-0.065 (0.043)	0.011 (0.083)	0.185 (0.173)	-0.052 (0.085)
* $p < 0.05$; ** $p < 0.001$						
Note: BACS: Brief Assessment of Cognition in Schizophrenia; HISOC: High Risk Social Skills; BDIT: Binocular Depth Inversion; WMSSS Wechsler Memory Scale - Spatial Span; SG: Snakes in the Grass; BABBLE: Babble Task; CPT: Continuous Performance Task						
MatStage: Maturational Stage - Median split of Age at baseline						
β0: Intercept; β(Time): Effect of Time; β(Time2); Non-linear Effect of Time; β(Group*Time*MatStage): Interaction/Differential Group & Maturational Effects over time						
β(Group*Time^2*MatStage): Non-linear Interaction/Differential Group & Maturational Effects Effects over time						

© 2018 Lam L et al. *JAMA Psychiatry.*

eTable 3 cont'd. Linear Mixed Models Elements for Maturational Stage investigation						
	$β_0$	β(Time)	β($Time^2$)	β(MatStage)	β(Group*Time *MatStage)	β(Group*$Time^2$ *MatStage)
Healthy Controls, Remitters, and Non-Remitters						
BACS Verbal Memory	-0.026 (0.065)	**0.316 (0.1)***	-0.032 (0.049)	0.024 (0.077)	**0.341 (0.133)***	-0.117 (0.067)
BACS Digit Sequencing	-0.035 (0.069)	**0.256 (0.088)***	-0.055 (0.042)	0.039 (0.083)	0.33 (0.189)	0.034 (0.025)
BACS Token Motor Task	-0.041 (0.062)	**0.478 (0.099)****	**-0.196 (0.048)****	-0.005 (0.072)	-0.125 (0.13)	0.063 (0.066)
BACS Semantic Fluency (animals)	0.034 (0.069)	0.009 (0.089)	0.002 (0.044)	-0.066 (0.082)	0.166 (0.119)	-0.053 (0.059)
BACS Semantic Fluency (fruits)	0.031 (0.068)	-0.167 (0.101)	**0.112 (0.05)***	-0.062 (0.081)	**0.291 (0.134)***	**-0.154 (0.067)***
BACS Semantic Fluency (veg)	-0.028 (0.069)	**-0.207 (0.093)***	**0.111 (0.045)***	-0.005 (0.082)	0.077 (0.123)	-0.012 (0.062)
BACS Semantic Fluency (total)	0.019 (0.071)	-0.136 (0.074)	**0.083 (0.036)***	-0.055 (0.087)	**0.213 (0.1)***	-0.085 (0.049)
BACS Symbol Coding	-0.004 (0.07)	**0.308 (0.079)****	-0.069 (0.039)	-0.018 (0.086)	0.208 (0.106)	-0.071 (0.052)
BACS Tower of London	-0.038 (0.061)	**0.335 (0.111)***	-0.106 (0.055)	0.039 (0.067)	0.26 (0.145)	-0.093 (0.074)
HISOC Affect/Expression	-0.081 (0.075)	-0.236 (0.124)	0.046 (0.061)	0.102 (0.09)	-0.109 (0.187)	0.07 (0.095)
HISOC Behavior/Language	-0.08 (0.073)	0.165 (0.139)	-0.13 (0.069)	0.127 (0.085)	0.102 (0.207)	-0.021 (0.106)
HISOC Comm/Social	-0.053 (0.073)	-0.103 (0.127)	-0.015 (0.062)	0.089 (0.085)	-0.006 (0.189)	0.042 (0.097)
HISOC Average	-0.088 (0.074)	-0.072 (0.124)	-0.033 (0.061)	0.127 (0.088)	-0.011 (0.186)	0.035 (0.095)
* p < 0.05; ** p < 0.001						
Note: BACS: Brief Assessment of Cognition in Schizophrenia; HISOC: High Risk Social Skills; BDIT: Binocular Depth Inversion; WMSSS Wechsler Memory Scale - Spatial Span; SG: Snakes in the Grass; BABBLE: Babble Task; CPT: Continuous Performance Task						
MatStage: Maturational Stage - Median split of Age at baseline						
β0: Intercept; β(Time): Effect of Time; β(Time2); Non-linear Effect of Time; β(Group*Time*MatStage): Interaction/Differential Group & Maturational Effects over time						
β(Group*Time^2*MatStage): Non-linear Interaction/Differential Group & Maturational Effects Effects over time						

© 2018 Lam L et al. *JAMA Psychiatry.*

eTable 3 cont'd. Linear Mixed Models Elements for Maturational Stage investigation						
	β_0	β(Time)	β(Time^2)	β(MatStage)	β(Group*Time *MatStage)	β(Group*Time^2 *MatStage)
BDIT Total	0.02 (0.071)	**-0.238 (0.083)***	**0.093 (0.04)***	-0.011 (0.087)	-0.048 (0.113)	0.007 (0.055)
BDIT Total Index	0.017 (0.071)	**-0.227 (0.083)***	**0.09 (0.04)***	-0.005 (0.087)	-0.052 (0.113)	0.008 (0.055)
BDIT Faces	0.006 (0.071)	-0.156 (0.088)	0.059 (0.043)	0.017 (0.086)	-0.145 (0.118)	0.068 (0.058)
BDIT Faces Index	0.008 (0.071)	-0.155 (0.088)	0.058 (0.043)	0.014 (0.086)	-0.143 (0.118)	0.067 (0.058)
WMSIIIss	0.018 (0.063)	-0.015 (0.096)	0.065 (0.047)	-0.066 (0.075)	0.131 (0.127)	-0.052 (0.064)
SGTargetrt	-0.01 (0.065)	**0.682 (0.097)****	**-0.218 (0.047)****	-0.045 (0.075)	0.069 (0.128)	-0.025 (0.064)
SGTargetAcc	0.054 (0.064)	**0.284 (0.106)***	**-0.105 (0.052)***	-0.135 (0.07)	0.007 (0.139)	-0.004 (0.071)
SGDistractrt	-0.024 (0.066)	**0.687 (0.101)****	**-0.205 (0.049)****	-0.024 (0.077)	0.109 (0.134)	-0.055 (0.067)
SGDistractAcc	0.034 (0.063)	0.138 (0.106)	-0.023 (0.052)	-0.096 (0.071)	0.027 (0.139)	0.039 (0.071)
SGAllsnakesrt	0.022 (0.065)	**0.536 (0.117)****	**-0.21 (0.058)****	-0.071 (0.073)	0.095 (0.153)	-0.027 (0.078)
SGAllsnakesacc	0.022 (0.065)	**0.536 (0.117)****	**-0.21 (0.058)****	-0.071 (0.073)	0.095 (0.153)	-0.027 (0.078)
BABBLEphrase	0.018 (0.063)	**0.236 (0.106)***	-0.089 (0.052)	0.002 (0.071)	-0.112 (0.139)	0.077 (0.071)
BABBLEwords	0.026 (0.068)	**0.502 (0.088)****	**-0.182 (0.043)****	-0.033 (0.081)	-0.089 (0.117)	0.105 (0.059)
CPT2d	0.029 (0.066)	**0.248 (0.119)***	-0.104 (0.059)	-0.066 (0.076)	-0.006 (0.156)	0.038 (0.079)
CPT3d	-0.026 (0.069)	0.154 (0.105)	-0.027 (0.051)	0.047 (0.08)	0.043 (0.139)	0.009 (0.07)
CPT4d	-0.041 (0.065)	**0.271 (0.091)***	-0.078 (0.045)	0.009 (0.077)	0.146 (0.121)	-0.036 (0.061)
CPTaveraged	-0.034 (0.068)	**0.256 (0.086)***	-0.073 (0.042)	0.035 (0.082)	0.102 (0.115)	-0.011 (0.057)
* $p < 0.05$; ** $p < 0.001$						
Note: BACS: Brief Assessment of Cognition in Schizophrenia; HISOC: High Risk Social Skills; BDIT: Binocular Depth Inversion; WMSSS Wechsler Memory Scale - Spatial Span; SG: Snakes in the Grass; BABBLE: Babble Task; CPT: Continuous Performance Task						
MatStage: Maturational Stage - Median split of Age at baseline						
β0: Intercept; β(Time): Effect of Time; β(Time2); Non-linear Effect of Time; β(Group*Time*MatStage): Interaction/Differential Group & Maturational Effects over time						
β(Group*Time^2*MatStage): Non-linear Interaction/Differential Group & Maturational Effects Effects over time						

**eTable 4**. Linear Mixed Model Elements for UHR Ascertainment Age and Age-Related Trajectories						
	β_0	β(Time)	β($Time^2$)	β(UHR Age)	β(Group*Time*UHR Age*Age)	β(Group*$Time^2$*UHR Age*Age)
Healthy Controls, Non-converters, and Converters						
BACS Verbal Memory	-0.027(0.049)	**0.343(0.117)***	-0.04(0.049)	0.025(0.059)	**0.012(0.006)***	-0.005(0.003)
BACS Digit Sequencing	-0.019(0.052)	0.144(0.11)	-0.062(0.046)	-0.105(0.057)	0.001(0.005)	0.002(0.003)
BACS Token Motor Task	-0.015(0.047)	**0.409(0.115)****	**-0.17(0.048)****	-0.021(0.058)	0.005(0.006)	-0.001(0.003)
BACS Semantic Fluency (animals)	0(0.051)	0.061(0.104)	-0.011(0.043)	0.017(0.054)	0.005(0.005)	-0.001(0.003)
BACS Semantic Fluency (fruits)	0.015(0.051)	**-0.273(0.118)***	0.097(0.05)	**-0.121(0.06)***	0.011(0.006)	-0.005(0.003)
BACS Semantic Fluency (veg)	-0.008(0.052)	**-0.32(0.108)***	**0.143(0.045)***	-0.042(0.056)	**0.014(0.005)***	-0.005(0.003)
BACS Semantic Fluency (total)	0.004(0.053)	**-0.18(0.087)***	**0.083(0.036)***	-0.042(0.047)	**0.011(0.004)***	-0.004(0.002)
BACS Symbol Coding	-0.02(0.052)	**0.272(0.093)***	**-0.086(0.038)***	-0.075(0.05)	0.006(0.005)	-0.002(0.002)
BACS Tower of London	-0.018(0.046)	**0.514(0.128)****	**-0.144(0.055)***	**0.122(0.062)***	0.003(0.006)	0(0.003)
HISOC Affect/Expression	-0.017(0.056)	-0.256(0.145)	0.067(0.061)	0.028(0.072)	0.004(0.008)	0(0.004)
HISOC Behavior/Language	-0.011(0.055)	0.173(0.161)	-0.115(0.069)	0.045(0.079)	0.009(0.009)	-0.004(0.004)
HISOC Comm/Social	-0.016(0.056)	-0.077(0.147)	-0.017(0.062)	0.016(0.072)	-0.003(0.008)	0.002(0.004)
HISOC Average	-0.02(0.056)	-0.062(0.144)	-0.023(0.061)	0.035(0.071)	0.003(0.008)	0(0.004)
* $p < 0.05$; ** $p < 0.001$						
Note: BACS: Brief Assessment of Cognition in Schizophrenia; HISOC: High Risk Social Skills; BDIT: Binocular Depth Inversion; WMSSS Wechsler Memory Scale - Spatial Span; SG: Snakes in the Grass; BABBLE: Babble Task; CPT: Continuous Performance Task						
UHR Age: Age of Ascertainment of UHR status						
β0: Intercept; β(Time): Effect of Time; β(Time2); Non-linear Effect of Time; β(Group*Time*UHR Age*Age): Interaction/Differential Group & Age Trajectory Effects over time						
β(Group*Time2*UHR Age*Age): Non-linear Interaction/Differential Group & Age Trajectory Effects over time						
Highlighted models were selected for post-hoc testing						

© 2018 Lam L et al. *JAMA Psychiatry.*

eTable 4 cont'd. Linear Mixed Model Elements for UHR Ascertainment Age and Age-Related Trajectories						
	β_0	β(Time)	β(Time^2)	β(UHR Age)	β(Group*Time*UHR Age*Age)	β(Group*Time^2*UHR Age*Age)
Healthy Controls, Non-converters, and Converters						
BDIT Total	0.019(0.053)	-0.182(0.098)	**0.088(0.04)***	0.045(0.053)	0(0.005)	0(0.002)
BDIT Total Index	0.019(0.053)	-0.175(0.098)	**0.085(0.04)***	0.042(0.053)	0(0.005)	0(0.002)
BDIT Faces	0.027(0.053)	-0.043(0.103)	0.054(0.042)	0.102(0.055)	-0.005(0.005)	0.002(0.003)
BDIT Faces Index	0.027(0.053)	-0.043(0.103)	0.052(0.042)	0.097(0.055)	-0.005(0.005)	0.003(0.003)
WMSIIIss	-0.021(0.048)	0.01(0.112)	0.052(0.047)	-0.012(0.056)	0.003(0.006)	-0.001(0.003)
SGTargetrt	-0.03(0.049)	**0.646(0.113)****	**-0.202(0.047)****	0.001(0.058)	0.011(0.006)	-0.004(0.003)
SGTargetAcc	0.011(0.049)	**0.289(0.123)***	-0.092(0.052)	0.004(0.061)	0.006(0.006)	-0.002(0.003)
SGDistractrt	-0.032(0.05)	**0.652(0.119)****	**-0.193(0.049)****	0.007(0.062)	0.011(0.006)	-0.004(0.003)
SGDistractAcc	-0.014(0.048)	0.054(0.124)	-0.004(0.052)	-0.066(0.063)	0.007(0.006)	-0.001(0.003)
SGAllsnakesrt	0.009(0.049)	**0.472(0.135)***	**-0.162(0.057)***	0.033(0.067)	**0.021(0.007)***	**-0.008(0.003)***
SGAllsnakesacc	0.009(0.049)	**0.472(0.135)***	**-0.162(0.057)***	0.033(0.067)	**0.021(0.007)***	**-0.008(0.003)***
BABBLEphrase	-0.011(0.049)	0.114(0.123)	-0.064(0.052)	-0.075(0.062)	-0.003(0.006)	0.001(0.003)
BABBLEwords	-0.022(0.051)	**0.454(0.103)****	**-0.168(0.043)****	-0.032(0.054)	-0.004(0.005)	0.003(0.003)
CPT2d	0.021(0.05)	0.03(0.138)	-0.035(0.058)	-0.088(0.068)	**0.02(0.007)***	**-0.008(0.003)***
CPT3d	0.013(0.051)	0.07(0.122)	0.02(0.051)	0.011(0.063)	**0.015(0.006)***	**-0.007(0.003)***
CPT4d	-0.028(0.048)	0.194(0.105)	-0.055(0.044)	-0.023(0.053)	**0.014(0.005)***	-0.006(0.003)
CPTaveraged	-0.005(0.05)	0.163(0.1)	-0.035(0.042)	-0.013(0.052)	**0.015(0.005)***	**-0.004(0.003)***
* p < 0.05; ** p < 0.001						
Note: BACS: Brief Assessment of Cognition in Schizophrenia; HISOC: High Risk Social Skills; BDIT: Binocular Depth Inversion; WMSSS Wechsler Memory Scale - Spatial Span; SG: Snakes in the Grass; BABBLE: Babble Task; CPT: Continuous Performance Task						
UHR Age: Age of Ascertainment of UHR status						
β0: Intercept; β(Time): Effect of Time; β(Time2); Non-linear Effect of Time; β(Group*Time*UHR Age*Age): Interaction/Differential Group & Age Trajectory Effects over time						
β(Group*Time2*UHR Age*Age): Non-linear Interaction/Differential Group & Age Trajectory Effects over time						
Highlighted models were selected for post-hoc testing						

© 2018 Lam L et al. *JAMA Psychiatry.*

eTable 4 cont'd. Linear Mixed Model Elements for UHR Ascertainment Age and Age-Related Trajectories						
	$β_0$	β(Time)	β(Time^2)	β(UHR Age)	β(Group*Time*UHR Age*Age)	β(Group*Time^2*UHR Age*Age)
Healthy Controls, Remitters, and Non-Remitters						
BACS Verbal Memory	-0.024(0.049)	**0.318(0.115)***	-0.028(0.048)	0.024(0.059)	**0.011(0.004)***	**-0.005(0.002)***
BACS Digit Sequencing	-0.017(0.052)	0.119(0.108)	-0.049(0.045)	-0.101(0.057)	0.003(0.004)	0.001(0.002)
BACS Token Motor Task	-0.028(0.047)	**0.366(0.113)***	**-0.156(0.047)***	-0.028(0.058)	0.005(0.004)	-0.002(0.002)
BACS Semantic Fluency (animals)	-0.004(0.051)	0.051(0.103)	-0.007(0.043)	0.017(0.054)	0.004(0.004)	-0.001(0.002)
BACS Semantic Fluency (fruits)	-0.001(0.051)	**-0.283(0.117)***	**0.099(0.049)***	**-0.124(0.06)***	**0.008(0.004)***	-0.003(0.002)
BACS Semantic Fluency (veg)	-0.025(0.052)	**-0.299(0.107)***	**0.132(0.044)***	-0.042(0.056)	**0.008(0.004)***	-0.002(0.002)
BACS Semantic Fluency (total)	-0.011(0.053)	**-0.172(0.086)***	**0.079(0.036)***	-0.041(0.047)	**0.007(0.003)***	-0.002(0.001)
BACS Symbol Coding	-0.02(0.052)	**0.269(0.092)***	**-0.086(0.038)***	-0.072(0.05)	0.003(0.003)	-0.001(0.002)
BACS Tower of London	-0.02(0.046)	**0.511(0.126)****	**-0.146(0.054)***	0.12(0.061)	0.002(0.004)	0.001(0.002)
HISOC Affect/Expression	-0.016(0.056)	-0.244(0.143)	0.052(0.061)	0.012(0.071)	0(0.006)	0.002(0.003)
HISOC Behavior/Language	-0.013(0.055)	0.169(0.16)	-0.124(0.068)	0.024(0.079)	0.004(0.006)	-0.002(0.003)
HISOC Comm/Social	-0.012(0.055)	-0.083(0.145)	-0.024(0.062)	-0.001(0.072)	-0.003(0.006)	0.002(0.003)
HISOC Average	-0.019(0.055)	-0.059(0.142)	-0.035(0.06)	0.016(0.071)	-0.001(0.006)	0.001(0.003)
BDIT Total	0.02(0.052)	**-0.202(0.098)***	**0.096(0.039)***	0.045(0.053)	0(0.003)	0(0.002)
* p < 0.05; ** p < 0.001						
Note: BACS: Brief Assessment of Cognition in Schizophrenia; HISOC: High Risk Social Skills; BDIT: Binocular Depth Inversion; WMSSS Wechsler Memory Scale - Spatial Span; SG: Snakes in the Grass; BABBLE: Babble Task; CPT: Continuous Performance Task						
UHR Age: Age of Ascertainment of UHR status						
β0: Intercept; β(Time): Effect of Time; β(Time2); Non-linear Effect of Time; β(Group*Time*UHR Age*Age): Interaction/Differential Group & Age Trajectory Effects over time						
β(Group*Time2*UHR Age*Age): Non-linear Interaction/Differential Group & Age Trajectory Effects over time						
Highlighted models were selected for post-hoc testing						

© 2018 Lam L et al. *JAMA Psychiatry.*

eTable 4 cont'd. Linear Mixed Model Elements for UHR Ascertainment Age and Age-Related Trajectories						
	β_0	β(Time)	β($Time^2$)	β(UHR Age)	β(Group*Time*UHR Age*Age)	β(Group*$Time^2$*UHR Age*Age)
Healthy Controls, Remitters, and Non-Remitters						
BDIT Total Index	0.02(0.052)	**-0.196(0.098)***	**0.094(0.039)***	0.042(0.053)	0(0.003)	0(0.002)
BDIT Faces	0.022(0.053)	-0.077(0.102)	0.07(0.042)	0.103(0.055)	-0.002(0.003)	0.001(0.002)
BDIT Faces Index	0.021(0.053)	-0.077(0.102)	0.067(0.042)	0.098(0.055)	-0.002(0.003)	0.001(0.002)
WMSIIIss	-0.019(0.047)	-0.016(0.11)	0.062(0.046)	-0.015(0.056)	0.004(0.004)	-0.001(0.002)
SGTargetrt	-0.023(0.049)	**0.632(0.111)****	**-0.194(0.046)****	0.004(0.058)	**0.008(0.004)***	-0.003(0.002)
SGTargetAcc	-0.007(0.049)	**0.299(0.121)***	**-0.103(0.051)***	-0.007(0.061)	0.002(0.004)	0(0.002)
SGDistractrt	-0.029(0.05)	**0.657(0.117)****	**-0.195(0.048)****	0.009(0.062)	0.007(0.004)	-0.003(0.002)
SGDistractAcc	-0.022(0.048)	0.077(0.122)	-0.014(0.051)	-0.064(0.063)	0.002(0.004)	0(0.002)
SGAllsnakesrt	-0.001(0.049)	**0.498(0.134)****	**-0.17(0.056)***	0.04(0.067)	**0.013(0.004)***	**-0.005(0.002)***
SGAllsnakesacc	-0.001(0.049)	**0.498(0.134)****	**-0.17(0.056)***	0.04(0.067)	**0.013(0.004)***	**-0.005(0.002)***
BABBLEphrase	0.011(0.048)	0.141(0.122)	-0.079(0.051)	-0.075(0.062)	-0.003(0.004)	0.002(0.002)
BABBLEwords	-0.004(0.051)	**0.471(0.102)****	**-0.174(0.042)****	-0.028(0.054)	-0.003(0.003)	0.002(0.002)
CPT2d	0.003(0.05)	0.043(0.136)	-0.047(0.058)	-0.096(0.068)	**0.012(0.005)***	-0.004(0.002)
CPT3d	0.004(0.051)	0.088(0.121)	0.004(0.05)	0.004(0.062)	**0.008(0.004)***	-0.003(0.002)
CPT4d	-0.032(0.048)	**0.255(0.104)***	-0.083(0.044)	-0.016(0.053)	0.005(0.004)	0(0.002)
CPTaveraged	-0.014(0.05)	**0.206(0.099)***	-0.059(0.041)	-0.014(0.052)	0.006(0.003)	-0.002(0.002)
* p < 0.05; ** p < 0.001						
Note: BACS: Brief Assessment of Cognition in Schizophrenia; HISOC: High Risk Social Skills; BDIT: Binocular Depth Inversion; WMSSS Wechsler Memory Scale - Spatial Span; SG: Snakes in the Grass; BABBLE: Babble Task; CPT: Continuous Performance Task						
UHR Age: Age of Ascertainment of UHR status						
β0: Intercept; β(Time): Effect of Time; β(Time2); Non-linear Effect of Time; β(Group*Time*UHR Age*Age): Interaction/Differential Group & Age Trajectory Effects over time						
β(Group*Time2*UHR Age*Age): Non-linear Interaction/Differential Group & Age Trajectory Effects over time						
Highlighted models were selected for post-hoc testing						

© 2018 Lam L et al. *JAMA Psychiatry.*

**eTable 5.** Post-Hoc Linear Mixed Model for UHR Ascertainment Age and Age-Related Trajectories						
	β_0	β(Time)	β(Time^2)	β(UHR Age)	β(Time*UHR Age*Age)	β(Time^2*UHR Age*Age)
Healthy Controls						
BACS Verbal Memory	-0.017(0.05)	0.166(0.189)	0.051(0.083)	0.102(0.083)	0.013(0.007)	-0.005(0.004)
BACS Semantic Fluency (total)	-0.004(0.051)	-0.331(0.136)*	0.14(0.058)*	-0.053(0.063)	0.005(0.005)	-0.002(0.003)
SGAllsnakesrt	-0.008(0.05)	0.172(0.217)	-0.102(0.096)	-0.037(0.094)	0.023(0.008)*	-0.008(0.004)
SGAllsnakesacc	-0.008(0.05)	0.172(0.217)	-0.102(0.096)	-0.037(0.094)	0.023(0.008)*	-0.008(0.004)
CPT2d	-0.005(0.051)	-0.085(0.213)	0.05(0.094)	0.035(0.091)	0.012(0.008)	-0.003(0.004)
Converters						
BACS Verbal Memory	-0.881(0.268)*	-0.406(0.607)	0.152(0.292)	-0.303(0.16)	0.01(0.048)	-0.002(0.024)
BACS Semantic Fluency (total)	-1.13(0.301)*	-0.138(0.363)	0.001(0.167)	-0.057(0.159)	0.004(0.028)	0.006(0.014)
SGAllsnakesrt	-0.499(0.247)	-0.851(0.872)	0.597(0.42)	0.226(0.255)	0.145(0.068)*	-0.07(0.034)
SGAllsnakesacc	-0.499(0.247)	-0.851(0.872)	0.597(0.42)	0.226(0.255)	0.145(0.068)*	-0.07(0.034)
CPT2d	-0.939(0.305)*	0.586(0.871)	-0.378(0.416)	-0.11(0.274)	-0.01(0.068)	0.005(0.034)
Non-converters						
BACS Verbal Memory	-0.441(0.084)**	0.515(0.185)*	-0.037(0.078)	0.075(0.101)	0.013(0.007)	-0.005(0.003)
BACS Semantic Fluency (total)	-0.293(0.1)*	-0.06(0.144)	0.071(0.06)	-0.026(0.081)	0.009(0.005)	-0.004(0.003)
SGAllsnakesrt	-0.105(0.088)	0.376(0.209)	-0.057(0.09)	0.088(0.111)	0.026(0.007)**	-0.01(0.004)*
SGAllsnakesacc	-0.105(0.088)	0.376(0.209)	-0.057(0.09)	0.088(0.111)	0.026(0.007)**	-0.01(0.004)*
CPT2d	-0.242(0.085)*	-0.009(0.223)	-0.064(0.096)	-0.161(0.118)	0.02(0.008)*	-0.006(0.004)
* $p < 0.05$; ** $p < 0.001$						
Note: BACS: Brief Assessment of Cognition in Schizophrenia; HISOC: High Risk Social Skills; BDIT: Binocular Depth Inversion; WMSSS Wechsler Memory Scale - Spatial Span; SG: Snakes in the Grass; BABBLE: Babble Task; CPT: Continuous Performance Task						
UHR Age: Age of Ascertainment of UHR status						
β0: Intercept; β(Time): Effect of Time; β(Time2); Non-linear Effect of Time; β(Time*UHR Age*Age): Interaction/Differential Age Trajectory Effects over time						
β(Time2*UHR Age*Age): Non-linear Interaction/Differential Age Trajectory Effects over time						

© 2018 Lam L et al. *JAMA Psychiatry.*

eTable 5 cont'd. Post-hoc Linear Mixed Model for UHR Ascertainment Age and Age-Related Trajectories						
	β_0	β(Time)	β($Time^2$)	β(UHR Age)	β(Time*UHR Age*Age)	β($Time^2$*UHR Age*Age)
Remitters						
BACS Verbal Memory	-0.278(0.116)*	0.426(0.216)*	0.022(0.088)	0.147(0.121)	0.009(0.008)	-0.001(0.004)
BACS Semantic Fluency (total)	-0.25(0.134)	-0.083(0.176)	0.08(0.072)	-0.028(0.102)	0.012(0.007)	-0.006(0.003)
SGAllsnakesrt	-0.185(0.115)	0.661(0.262)*	-0.165(0.11)	0.136(0.141)	0.026(0.009)*	-0.009(0.005)
SGAllsnakesacc	-0.185(0.115)	0.661(0.262)*	-0.165(0.11)	0.136(0.141)	0.026(0.009)*	-0.009(0.005)
CPT2d	-0.334(0.1)*	0.454(0.251)	-0.172(0.106)	0.042(0.135)	0.023(0.009)*	-0.008(0.005)
Non-Remitters						
BACS Verbal Memory	-0.72(0.103)**	0.322(0.289)	-0.123(0.135)	-0.289(0.116)*	0.02(0.011)	-0.011(0.006)
BACS Semantic Fluency (total)	-0.542(0.134)**	-0.064(0.209)	0.045(0.094)	-0.035(0.099)	0.005(0.008)	-0.001(0.004)
SGAllsnakesrt	-0.157(0.12)	-0.138(0.312)	0.191(0.145)	0.07(0.127)	0.034(0.011)*	-0.016(0.006)*
SGAllsnakesacc	-0.157(0.12)	-0.138(0.312)	0.191(0.145)	0.07(0.127)	0.034(0.011)*	-0.016(0.006)*
CPT2d	-0.293(0.133)*	-0.597(0.367)	0.039(0.17)	-0.471(0.156)*	0.019(0.013)	-0.006(0.007)
* $p < 0.05$; ** $p < 0.001$						
Note: BACS: Brief Assessment of Cognition in Schizophrenia; HISOC: High Risk Social Skills; BDIT: Binocular Depth Inversion; WMSSS Wechsler Memory Scale - Spatial Span; SG: Snakes in the Grass; BABBLE: Babble Task; CPT: Continuous Performance Task						
UHR Age: Age of Ascertainment of UHR status						
β0: Intercept; β(Time): Effect of Time; β(Time2); Non-linear Effect of Time; β(Time*UHR Age*Age): Interaction/Differential Age Trajectory Effects over time						
β(Time2*UHR Age*Age): Non-linear Interaction/Differential Age Trajectory Effects over time						

© 2018 Lam L et al. *JAMA Psychiatry.*

eTable 6. Marginal Homogeneity Test for Effect Distribution at Baseline Versus Follow-Up

	HC (BL vs FU)	UHRR (BL vs FU)	UHRNR (BL vs FU)	UHRNC (BL vs FU)	UHRC (BL vs FU)
Marginal Homogeneity	-4.43	-9.53	-16.30	-15.19	-23.62
SD	0.83	1.05	1.30	1.21	1.53
Z	-3.26	-3.61	-3.77	-3.55	-3.66
P	.00113	.000310	.000162	.000384	.000255
Adjusted P	.00563	.00155	.000808	.00192	.00127

Note: HC: Healthy Controls; UHRR: Remitters; UHRNR: Non-Remitters; UHRNC: Non-Converters; UHRC:Converters

BL: Baseline FU: Follow-up

© 2018 Lam L et al. *JAMA Psychiatry.*

eTable 7. Kolmogorov-Smirnov Tests for Component Loading Comparisons

	HC-UHRR (BL)	HC-UHRNR (BL)	UHRR-UHRNR (BL)	HC-UHRR (FU)	HC-UHRNR (FU)	UHRR-UHRNR (FU)	HC (BL vs FU)	UHRR (BL vs FU)	UHRNR (BL vs FU)
Dmax									
Social Cognition	0.53	0.18	0.59	0.18	0.35	0.41	0.29	0.35	0.53
Attention	0.18	0.35	0.35	0.24	0.35	0.29	0.24	0.29	0.24
Fluency	0.35	0.41	0.24	0.18	0.53	0.53	0.24	0.41	0.41
GCF	0.18	0.65	0.65	0.29	0.71	0.71	0.18	0.35	0.59
Perception	0.71	0.71	0.76	0.71	0.71	0.76	0.47	0.53	0.18
Chi-Square									
Social Cognition	5.85	0.00	8.03	0.00	1.35	2.52	0.53	1.35	5.85
Attention	0.00	1.35	1.35	0.10	1.35	0.53	0.10	0.53	0.10
Fluency	1.35	2.52	0.10	0.00	5.85	5.85	0.10	2.52	2.52
GCF	0.00	10.56	10.56	0.53	13.50	13.50	0.00	1.35	8.03
Perception	13.50	13.50	16.88	13.50	13.50	16.88	4.02	5.85	0.00
P-value									
Social Cognition	***.016***	.963	**.005**	.963	.245	.112	.465	.245	***.016***
Attention	.963	.245	.245	.751	.245	.465	.751	.465	.751
Fluency	.245	.112	.751	.963	***.016***	***.016***	.751	.112	.112
GCF	.963	**.001**	**.001**	.465	**.000239**	**.000239**	.963	.245	**.005**
Perception	**.000239**	**.000239**	**.0000397**	**.000239**	**.000239**	**.0000397**	*.045*	***.016***	.963

Note: GCF: General Cognitive Function.

Bold and underlined p-values survived Bonferroni Correction across 9 sets of comparisons, p-values in italics did not survive the correction

HC: Healthy Controls; UHRR: Remitters; UHRNR: Non-Remitters; BL: Baseline; FU: Follow-Up

© 2018 Lam L et al. *JAMA Psychiatry.*

eTable 8. Univariate One-Way ANOVA for Cognitive Components by Group Comparisons

	ANOVA		HC - UHRR (Post-Hoc)			HC - UHRNR (Post-Hoc)			UHRR - UHRNR (Post-Hoc)		
	F	P	Δ Mean	SE	P	Δ Mean	SE	P	Δ Mean	SE	P
zSocial_Cognition_WT_BL	20.32	3.08E-09	**.426***	.126	2.42E-03	**.749***	.126	1.66E-08	.323	.162	1.40E-01
zSocial_Cognition_WT_FU	20.61	2.51E-09	.315	.147	9.68E-02	**1.097***	.174	2.13E-09	**.781***	.212	7.70E-04
zSocial_Cognition_NWT_BL	17.70	3.57E-08	.275	.124	7.89E-02	**.720***	.124	2.93E-08	**.444***	.158	1.56E-02
zSocial_Cognition_NWT_FU	7.91	4.16E-04	.311	.133	5.88E-02	**.553***	.158	1.51E-03	.242	.192	6.26E-01
zAttention_WT_BL	32.01	7.17E-14	**.431***	.132	3.46E-03	**1.025***	.132	1.19E-13	**.594***	.169	1.45E-03
zAttention_WT_FU	12.95	3.28E-06	.109	.156	1.00E+00	**.941***	.185	1.53E-06	**.831***	.225	7.26E-04
zAttention_NWT_BL	7.36	7.04E-04	.291	.125	6.12E-02	**.427***	.125	2.05E-03	.136	.160	1.00E+00
zAttention_NWT_FU	2.86	5.80E-02	.157	.137	7.63E-01	.365	.163	7.68E-02	.208	.198	8.82E-01
zFluency_WT_BL	35.36	3.62E-15	**.418***	.133	5.10E-03	**1.091***	.133	4.06E-15	**.673***	.170	2.53E-04
zFluency_WT_FU	21.63	9.80E-10	.130	.152	1.00E+00	**1.184***	.180	3.64E-10	**1.055***	.219	5.86E-06
zFluency_NWT_BL	9.29	1.08E-04	.237	.127	1.87E-01	**.526***	.127	1.13E-04	.290	.162	2.25E-01
zFluency_NWT_FU	4.64	1.01E-02	.050	.137	1.00E+00	**.496***	.163	7.34E-03	.446	.198	7.47E-02
zGCF_WT_BL	22.48	4.14E-10	**.552***	.118	1.18E-05	**.660***	.118	1.18E-07	.108	.152	1.00E+00
zGCF_WT_FU	8.95	1.52E-04	.138	.132	8.87E-01	**.659***	.157	9.37E-05	**.521***	.191	1.96E-02
zGCF_NWT_BL	38.91	1.60E-16	**.509***	.123	1.20E-04	**1.033***	.123	1.15E-15	**.524***	.158	2.86E-03
zGCF_NWT_FU	20.37	3.12E-09	.102	.137	1.00E+00	**1.037***	.162	1.20E-09	**.935***	.198	8.82E-06
zPerception_WT_BL	3.63	2.73E-02	**.316***	.126	3.65E-02	.175	.126	4.92E-01	-.141	.161	1.00E+00
zPerception_WT_FU	3.66	2.65E-02	-.254	.135	1.83E-01	.269	.161	2.85E-01	**.523***	.195	2.32E-02
zPerception_NWT_BL	4.68	9.65E-03	.184	.123	4.03E-01	**.354***	.123	1.20E-02	.171	.157	8.35E-01
zPerception_NWT_FU	1.72	1.80E-01	-.109	.132	1.00E+00	.241	.157	3.77E-01	.350	.191	2.02E-01

Note: z: Standardized cognitive domain scores; Δ: mean difference; HC: Healthy Controls, UHRR: Remitters, UHRNR: Non-remitters

WT: Adjusted for cognitive factor structure changes over time NWT not adjusted for cognitive factor structure changes

* Significant Bonferonni corrected P-values

© 2018 Lam L et al. *JAMA Psychiatry.*

**eTable 9.** Repeated Measures ANOVA									
	ANOVA			HC paired t-test		UHRR paired t-test		UHRNR paired t-test	
	F (grp*time)	eta^2	P	t	P	t	P	t	P
Weighted									
Social Cognition	1.67	0.007	0.189	-	-	-	-	-	-
Attention	4.00	0.016	**0.019**	.145	.885	**-2.559**	**.013**	-.737	.464
Fluency	2.62	0.010	0.074	-	-	-	-	-	-
GCF	12.23	0.047	**7.00E-06**	.365	.715	**-4.281**	**5.64E-05**	-.768	.446
Perception	8.33	0.032	**2.77E-04**	.027	.978	**-4.690**	**1.26E-05**	-.423	.674
Unweighted									
Social Cognition	1.96	0.008	0.142	-	-	-	-	-	-
Attention	0.46	0.002	0.632	-	-	-	-	-	-
Fluency	1.67	0.007	0.189	-	-	-	-	-	-
GCF	9.72	0.038	**7.20E-05**	.383	.702	**-4.383**	**3.91E-05**	-.667	.508
Perception	3.60	0.140	0.028	-.001	1.000	-2.671	.009	-1.926	.060
Note: Weighted: Weighted for cognitive factor structure changes over time									
Unweighted: Not adjusted for cognitive factor structure changes over time<br>Values in boldface survives Bonferroni correction									

© 2018 Lam L et al. *JAMA Psychiatry.*

a.

b.

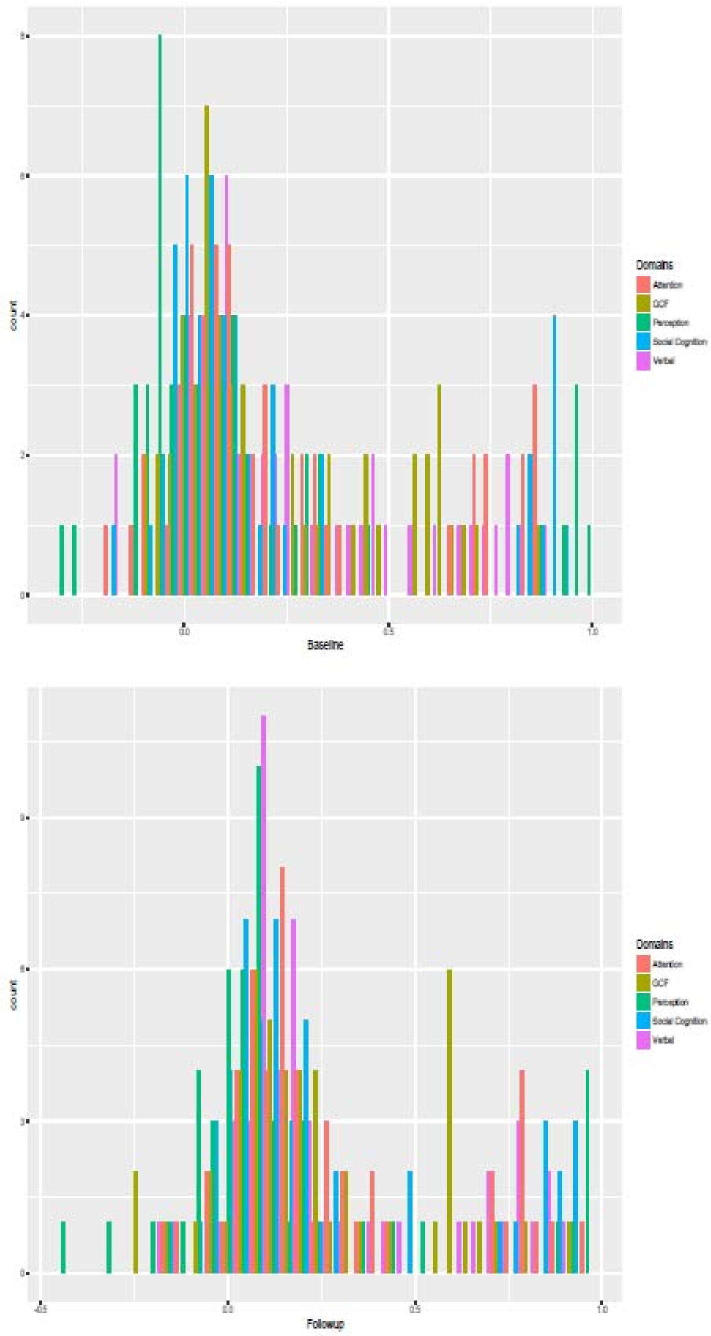

+

eFigure 1. Distribution of Factor Loadings for Baseline and Follow-Up
a. Baseline factor loadings b. Follow-up Factor Loadings.
GCF: General Cognitive Function

© 2018 Lam L et al. *JAMA Psychiatry.*

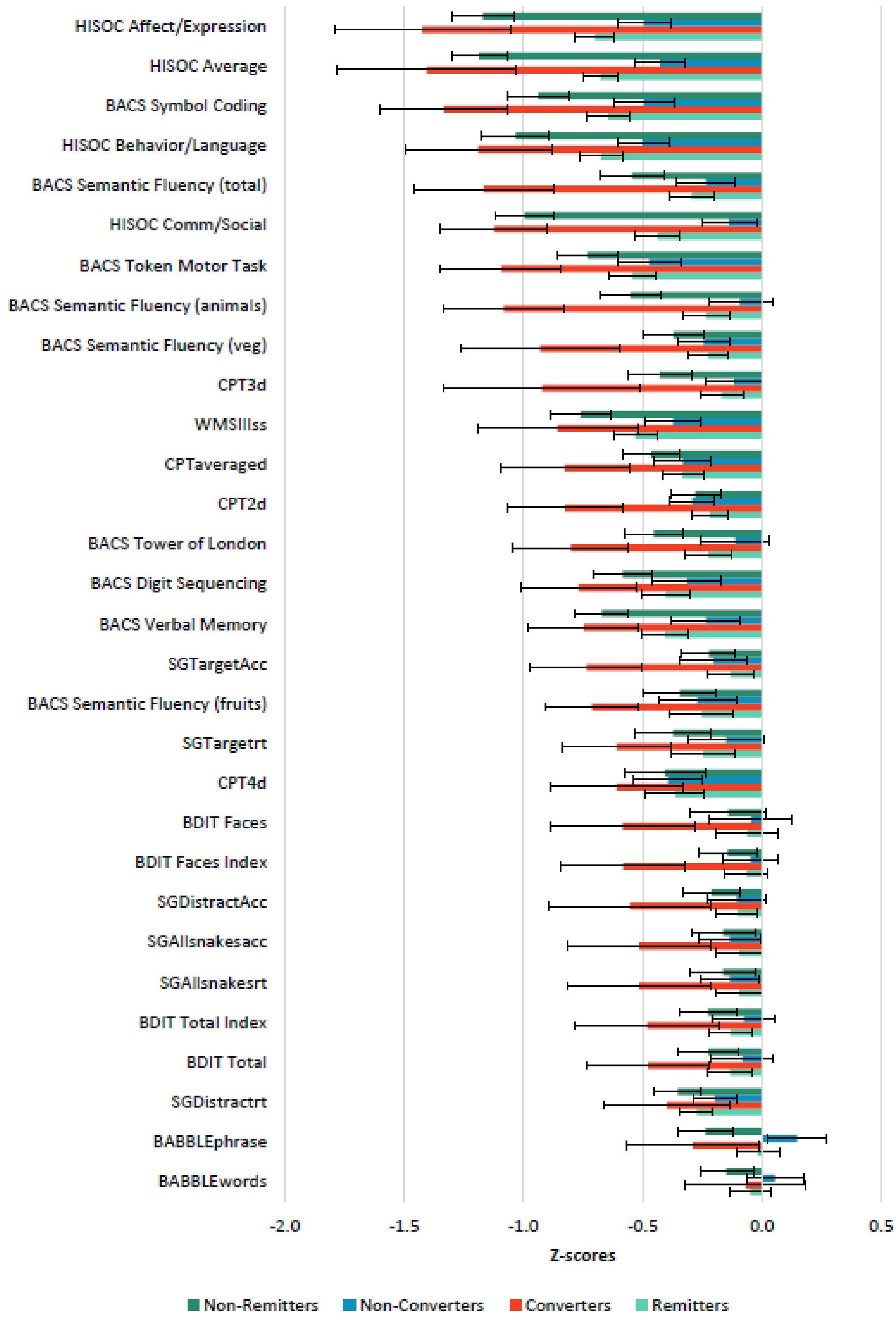

eFigure 2. Baseline Cognitive Profiles by Group

WMSIIIss: Wechsler Memory Scale III – Spatial Span; BACS: Brief Assessment of Cognition in Schizophrenia; BDIT:Binocular Depth Inversion Task; CPT: Continuous Performance Test; HISOC: High Risk Social Skills Interview; BABBLE: Babble Task; and SG: Snakes in the Grass. Tests are standardized with healthy controls are reference. Converters exists as a sub-set in the Non-Remitters group.

© 2018 Lam L et al. *JAMA Psychiatry.*

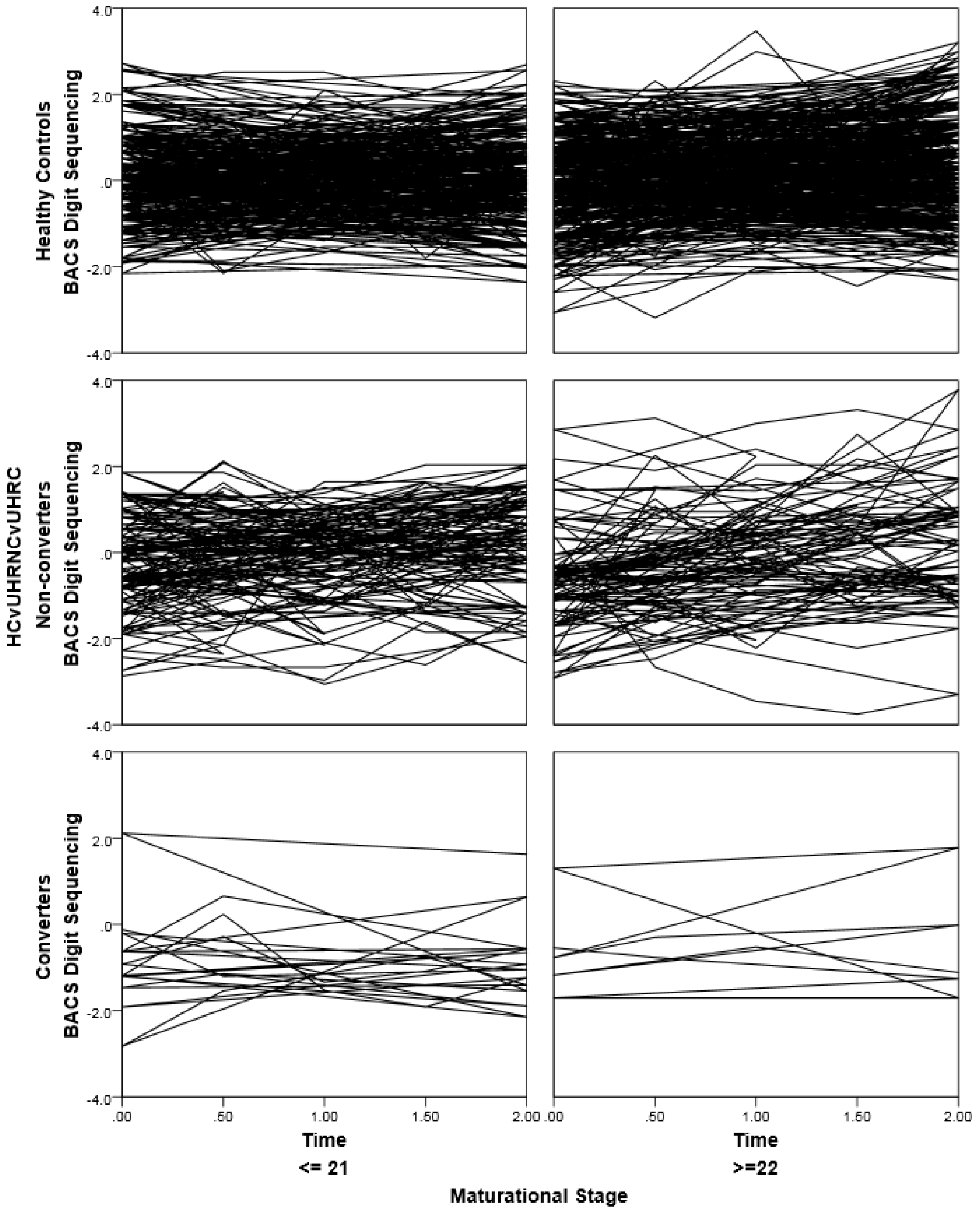

eFigure 3. BACS Digit Sequencing: Linear Mixed Model SPSS Path Plots by Healthy Controls, Non-Converters, Converters, Maturational Stage and Time

Post-hoc: HC: βAge Maturation*Time=0.498(0.104)*; UHRNC: βAge Maturation*Time=0.708(0.185)*; UHRC: βAge Maturation*Time=0.328(0.563)

* p < .05

** p < .001

© 2018 Lam L et al. *JAMA Psychiatry.*

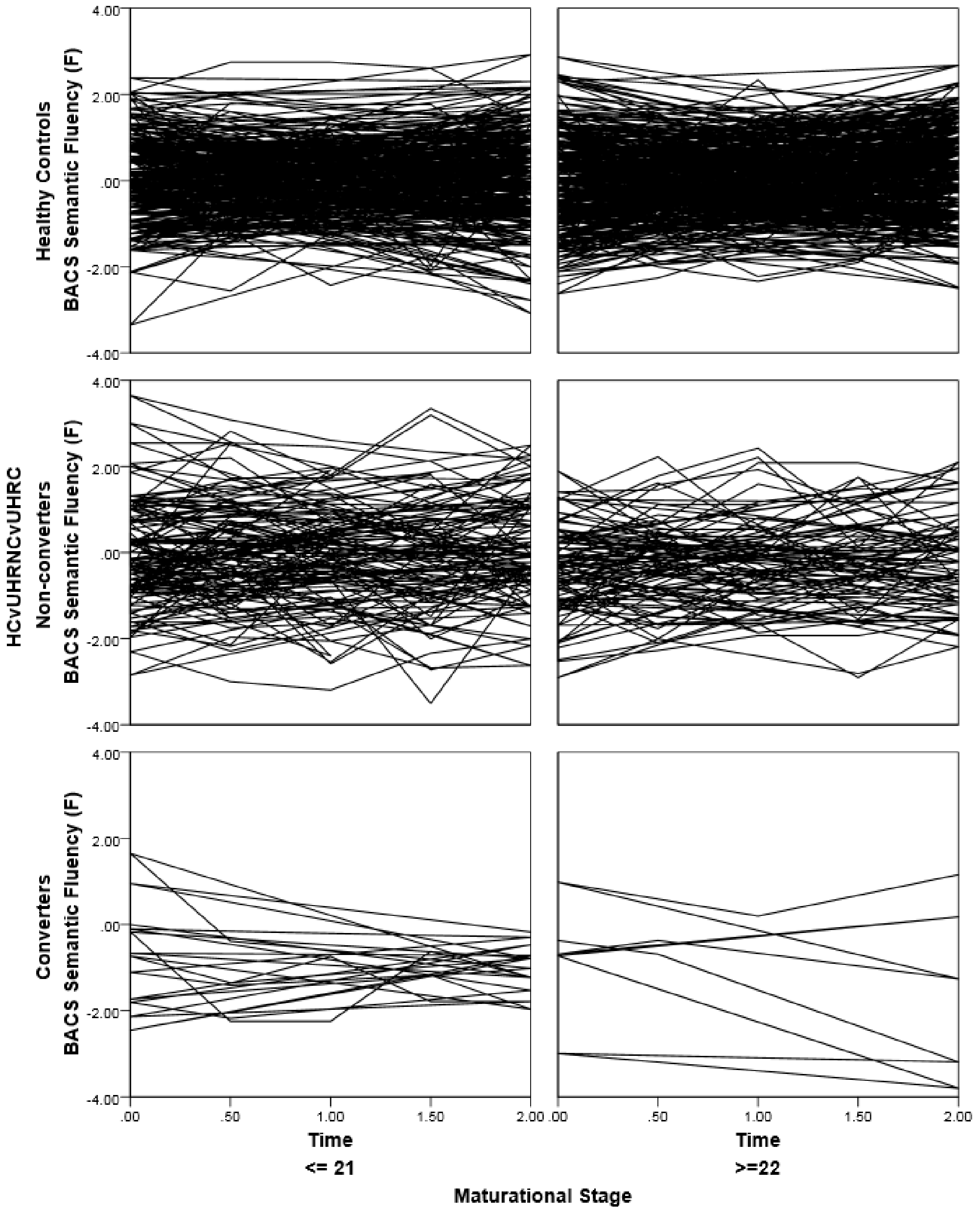

eFigure 4. BACS Semantic Fluency (F): Linear Mixed Model SPSS Path Plots by Healthy Controls, Non-converters, Converters, Maturational Stage and Time

Post-hoc: HC: βAge Maturation*Time=-0.003(0.101); UHRNC: βAge Maturation*Time=0.42(0.188); UHRC: βAge Maturation*Time=1.182(0.6)

* p < .05

** p < .001

© 2018 Lam L et al. *JAMA Psychiatry.*

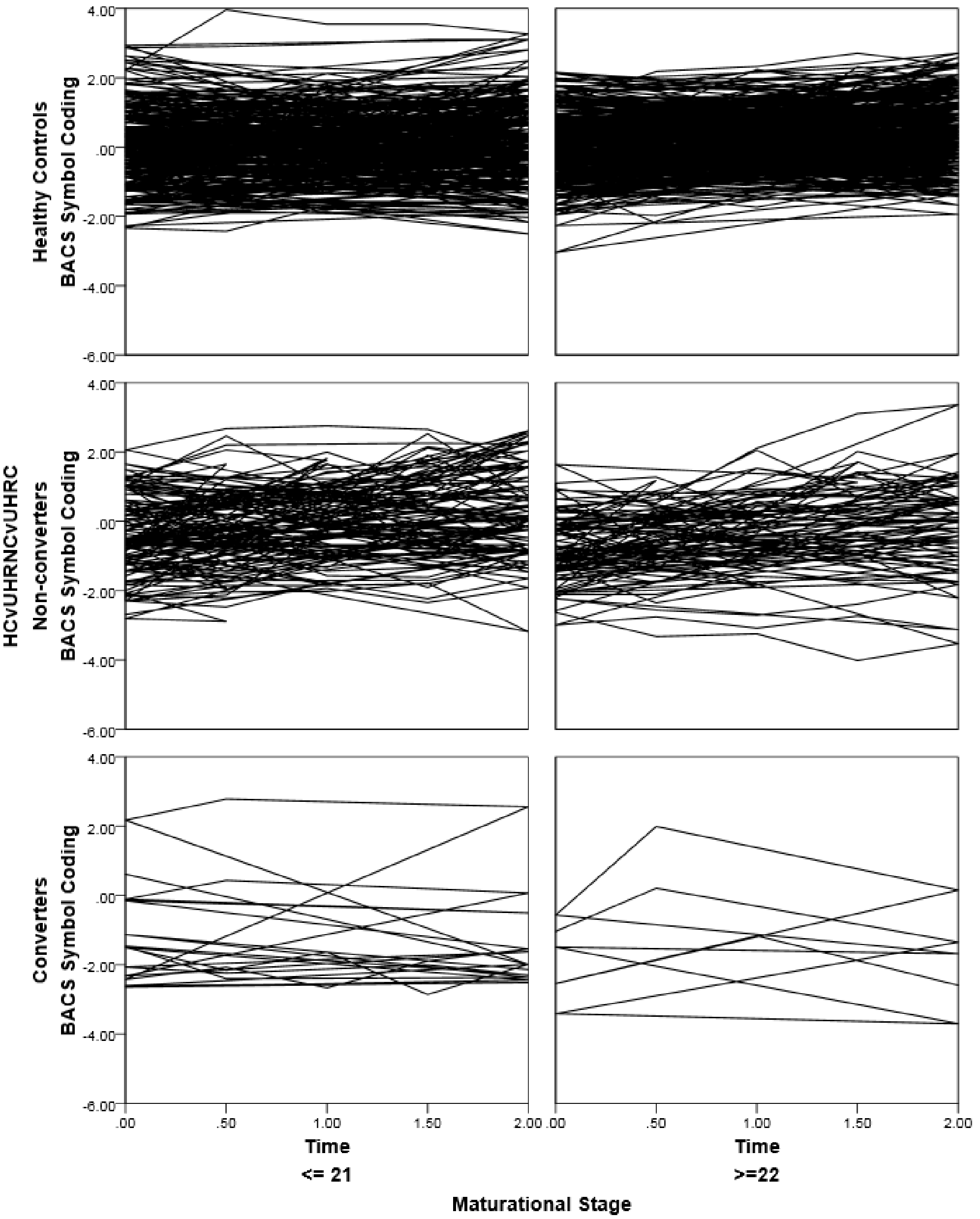

eFigure 5. BACS Symbol Coding: Linear Mixed Model SPSS Path Plots by Healthy Controls, Non-Converters, Converters, Maturational Stage and Time

Post-hoc: HC: βAge Maturation*Time=0.273(0.104); UHRNC: βAge Maturation*Time=0.026(0.173); UHRC: βAge Maturation*Time=4.298(0.759)**

* p < .05

** p < .001

© 2018 Lam L et al. *JAMA Psychiatry.*

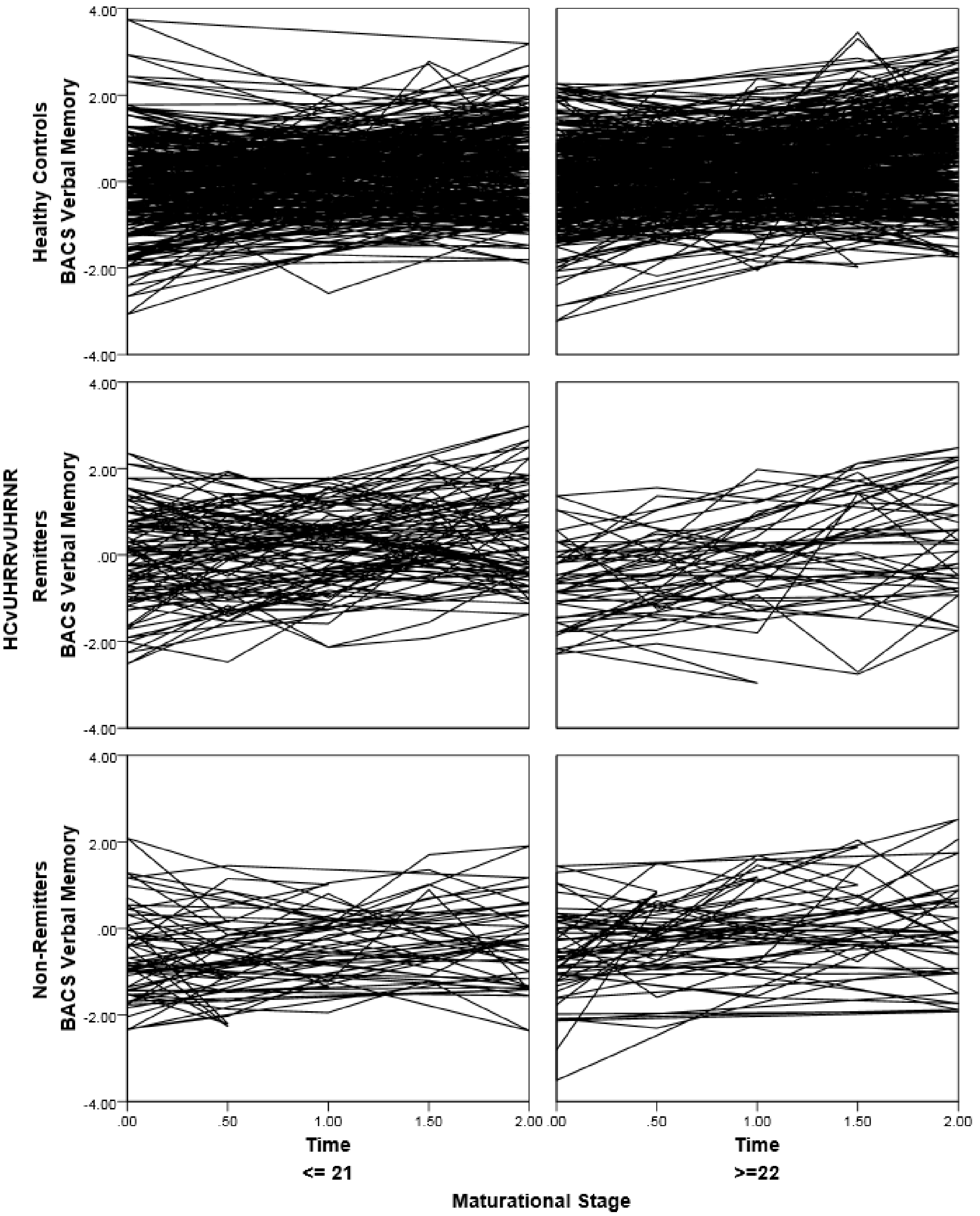

eFigure 6. BACS Verbal Memory: Linear Mixed Model SPSS Path Plots by Healthy Controls, Remitters, Non-Remitters, Maturational Stage and Time

Post-hoc: HC: βAge Maturation*Time=0.383(0.1); UHRR: βAge Maturation*Time=0.457(0.239); UHRNR: βAge Maturation*Time=0.691(0.21)

* $p < .05$

** $p < .001$

© 2018 Lam L et al. *JAMA Psychiatry.*

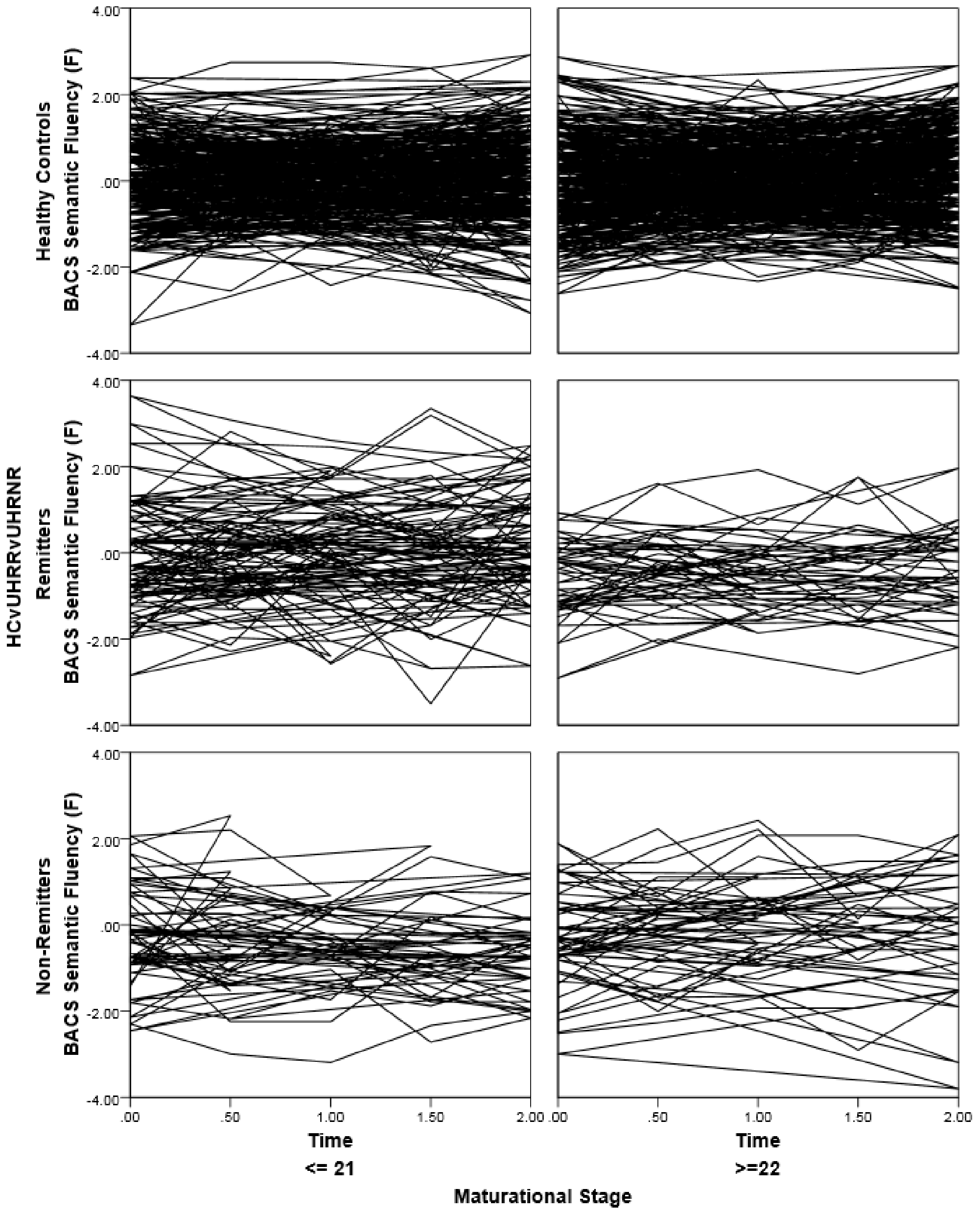

eFigure 7. BACS Semantic Fluency (F): Linear Mixed Model SPSS Path Plots by Healthy Controls, Remitters, Non-Remitters, Maturational Stage and Time

Post-hoc: HC: βAge Maturation*Time=-0.003(0.101); UHRR: βAge Maturation*Time=0.223(0.264); UHRNR: βAge Maturation*Time=0.882(0.249)*

* p < .05

** p < .001

© 2018 Lam L et al. *JAMA Psychiatry.*

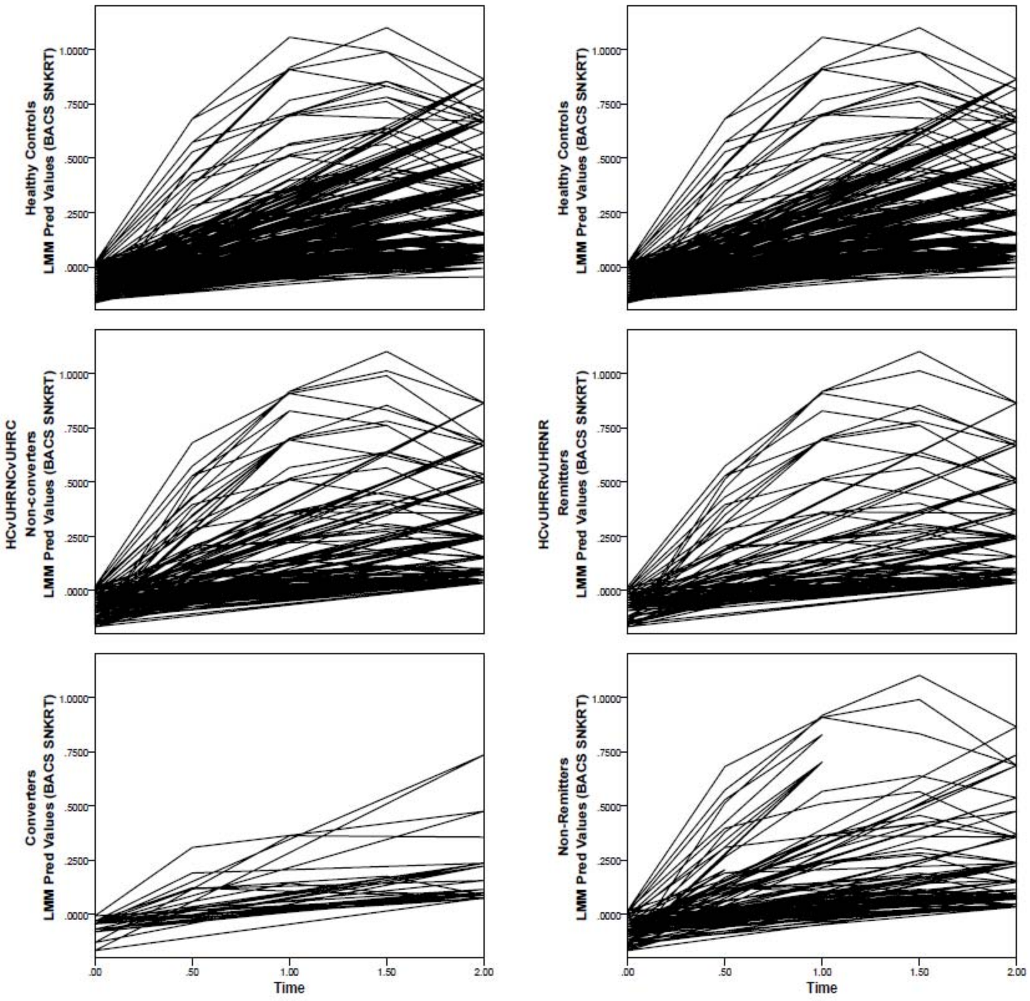

eFigure 8. Snakes in Grass (Reaction Time): Linear Mixed Model SPSS Path Plots of Predicted Score for Age Dependent Trajectories Across Groups

© 2018 Lam L et al. *JAMA Psychiatry.*

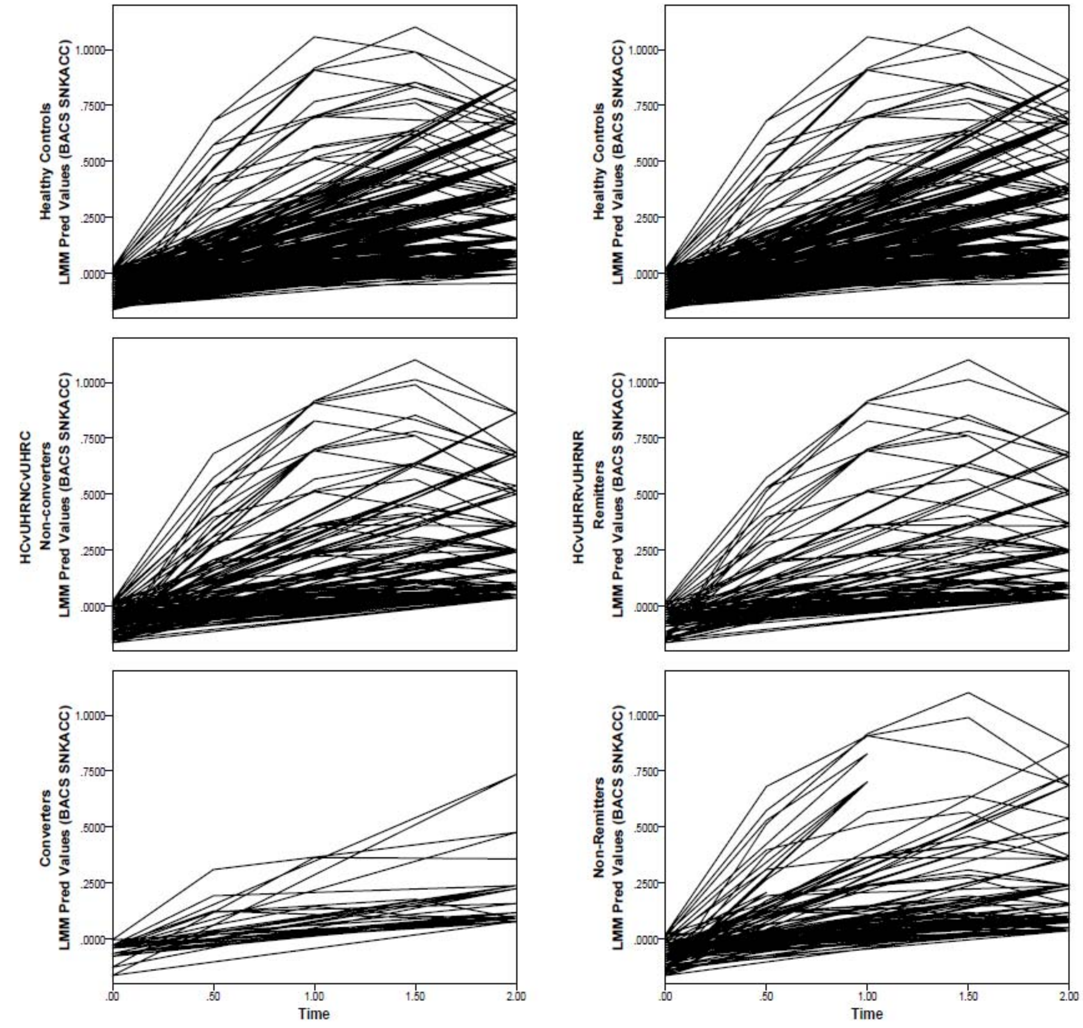

eFigure 9. Snakes in Grass (Accuracy): Linear Mixed Model SPSS Path Plots of Predicted Score for Age Dependent Trajectories Across Groups

© 2018 Lam L et al. *JAMA Psychiatry.*

References

1. Lorenzo-Seva U, Ten Berge JM. Tucker's congruence coefficient as a meaningful index of factor similarity. *Methodology*. 2006;2(2):57-64.

2. Diedenhofen B, Musch J. cocor: A Comprehensive Solution for the Statistical Comparison of Correlations. *PLOS ONE*. 2015;10(4):e0121945. doi:10.1371/journal.pone.0121945.

3. Tucker LR. *A Method for Synthesis of Factor Analysis Studies*. PN; 1951.

4. Davenport EC. Significance Testing of Congruence Coefficients: A Good Idea? *Educ Psychol Meas*. 1990;50(2):289-296. doi:10.1177/0013164490502007.

5. Paunonen SV. On Chance and Factor Congruence Following Orthogonal Procrustes Rotation. *Educ Psychol Meas*. 1997;57(1):33-59. doi:10.1177/0013164497057001003.

6. Diedenhofen B, Musch J. cocor: A comprehensive solution for the statistical comparison of correlations. *PloS One*. 2015;10(4):e0121945.

7. Bursac Z, Gauss CH, Williams DK, Hosmer DW. Purposeful selection of variables in logistic regression. *Source Code Biol Med*. 2008;3:17. doi:10.1186/1751-0473-3-17.

8. Sekhon JS. *Multivariate and Propensity Score Matching Software with Automated Balance Optimization: The Matching Package for R*. Rochester, NY: Social Science Research Network; 2008. https://papers.ssrn.com/abstract=1009044. Accessed February 21, 2018.

© 2018 Lam L et al. *JAMA Psychiatry.*
